# KLF13 restrains Dll4‐muscular Notch2 axis to improve the muscle atrophy

**DOI:** 10.1002/jcsm.13538

**Published:** 2024-07-08

**Authors:** Shu Yang, Lijiao Xiong, Guangyan Yang, Jiaqing Xiang, Lixing Li, Lin Kang, Zhen Liang

**Affiliations:** ^1^ Department of Geriatrics, The First Affiliated Hospital (Shenzhen People's Hospital) Southern University of Science and Technology Shenzhen China; ^2^ Guangdong Provincial Clinical Research Center for Geriatrics, Shenzhen Clinical Research Center for Geriatrics, The Second Clinical Medical College Jinan University (Shenzhen People's Hospital) Shenzhen China; ^3^ The Biobank of National Innovation Center for Advanced Medical Devices Shenzhen People's Hospital Shenzhen China

**Keywords:** Sarcopenia, KLF13, Notch, DLL4, Clofoctol

## Abstract

**Background:**

Muscle atrophy can cause muscle dysfunction and weakness. Krüppel‐like factor 13 (KLF13), a central regulator of cellular energy metabolism, is highly expressed in skeletal muscles and implicated in the pathogenesis of several diseases. This study investigated the role of KLF13 in muscle atrophy, which could be a novel therapeutic target.

**Methods:**

The effects of gene knockdown and pharmacological targeting of *KLF13* on skeletal muscle atrophy were investigated using cell‐based and animal models. Clofoctol, an antibiotic and KLF13 agonist, was also investigated as a candidate for repurposing. The mechanisms related to skeletal muscle atrophy were assessed by measuring the expression levels and activation statuses of key regulatory pathways and validated using gene knockdown and RNA sequencing.

**Results:**

In a dexamethasone‐induced muscle atrophy mouse model, the *KLF13* knockout group had decreased muscle strength (N) (1.77 ± 0.10 vs. 1.48 ± 0.16, *P* < 0.01), muscle weight (%) [gastrocnemius (Gas): 76.0 ± 5.69 vs. 60.7 ± 7.23, *P* < 0.001; tibialis anterior (TA): 75.8 ± 6.21 vs. 67.5 ± 5.01, *P* < 0.05], and exhaustive running distance (m) (495.5 ± 64.8 vs. 315.5 ± 60.9, *P* < 0.05) compared with the control group. *KLF13* overexpression preserved muscle mass (Gas: 100 ± 6.38 vs. 120 ± 14.4, *P* < 0.01) and the exhaustive running distance (423.8 ± 59.04 vs. 530.2 ± 77.45, *P* < 0.05) in an in vivo diabetes‐induced skeletal muscle atrophy model. Clofoctol treatment protected against dexamethasone‐induced muscle atrophy. Myotubes treated with dexamethasone, an atrophy‐inducing glucocorticoid, were aggravated by *KLF13* knockout, but anti‐atrophic effects were achieved by inducing *KLF13* overexpression. We performed a transcriptome analysis and luciferase reporter assays to further explore this mechanism, finding that delta‐like 4 (*Dll4*) was a novel target gene of KLF13. The KLF13 transcript repressed Dll4, inhibiting the Dll4‐Notch2 axis and preventing muscle atrophy. Dexamethasone inhibited *KLF13* expression by inhibiting myogenic differentiation 1 (i.e., MYOD1)‐mediated KLF13 transcriptional activation and promoting F‐Box and WD repeat domain containing 7 (i.e., FBXW7)‐mediated KLF13 ubiquitination.

**Conclusions:**

This study sheds new light on the mechanisms underlying skeletal muscle atrophy and potential drug targets. KLF13 regulates muscle atrophy and is a potential therapeutic target. Clofoctol is an attractive compound for repurposing studies to treat skeletal muscle atrophy.

## Introduction

Sarcopenia, an age‐related muscle disorder common among older adults, is a significant global concern, occurring in 10–27% of adults.^1^ Sarcopenia is characterized by the progressive loss of muscle mass and strength, leading to functional impairment and an increased risk of adverse events, falls, hospitalization and mortality, compromising the quality of life.^2^ Although muscle atrophy is often associated with conventional factors, such as aging and immobilization, its aetiology is intricate, with diverse contributors.^3^ The effects of chronic diseases on the subtle mechanisms of glucocorticoid (GC)‐induced muscle wasting and denervation‐induced muscle atrophy are insidious. Given this and the multifaceted nature of sarcopenia, investigating the molecular intricacies of muscle atrophy is essential since they may involve transcriptional regulation and protein degradation pathways, such as the ubiquitin–proteasome system and autophagy–lysosome pathway.^3^ Signalling pathways, such as myostatin/Smad and Notch/delta‐like 4 (Dll4), may contribute to sarcopenia.^3^, ^4^ Mitochondrial dysfunction is also associated with muscular atrophy, including impaired mitochondrial biogenesis, dynamics and energy production. Therefore, an in‐depth understanding of these intricate molecular mechanisms will enhance our understanding of the pathogenesis of sarcopenia and guide future prevention and treatment strategies.

Skeletal muscle is the major site for glycogen storage and amino acid preservation and is critical for maintaining whole‐body metabolic homeostasis.^5^ Krüppel‐like factors (KLFs) are a family of zinc finger transcription factors that play pivotal roles in various physiological and pathological processes, including cell proliferation, differentiation and tissue development. Accumulating evidence implicates KLFs in regulatory functions related to skeletal muscle development and metabolism.^5^ For instance, KLF3, KLF5 and KLF15 promote skeletal myoblast differentiation whereas KLF6 and KLF7 exert inhibitory effects on skeletal myogenesis.^5^, ^6^ KLF2 and KLF4 control muscle fusion in an extracellular signal‐regulated kinase 5 (i.e., ERK5), myogenic differentiation 1 (MYOD1) and myocyte enhancer factor 2 (i.e., MEF2) independent manner.^7^, ^8^ In mature skeletal muscle, KLF15 regulates lipid flux and systemic metabolic homeostasis,^9^, ^10^ and KLF5 cooperates with peroxisome proliferator‐activated receptor δ (i.e., PPARδ) to promote fatty acid transport and oxidation.^6^, ^10^ KLF family members, such as KLF5 and KLF15,^11^, ^12^ help regulate skeletal muscle atrophy, although the underlying mechanisms remain unclear.

KLF13 facilitates the differentiation of cardiac muscles and pre‐adipocytes.^13^, ^14^ Our previous study found that KLF13 recruits a repressor complex comprising SIN3A and histone deacetylase 1 (i.e., HDAC1) to the target genes of transforming growth factor‐beta (TGF‐β), limiting the profibrotic effects of TGF‐β.^15^ However, its role in skeletal muscle remains unknown. Therefore, this study explored the involvement of KLF13 in sarcopenia to elucidate the underlying molecular mechanisms and suggest novel therapeutic targets for mitigating muscle atrophy and improving the quality of life of individuals with sarcopenia.

## Methods

### Animal models

*Klf13* global knockout mice (B6/JGpt‐*Klf13* ^*em2Cd7440*^/Gpt; Strain ID: T014500) were purchased from Gempharmatech Co. Ltd (Jiangsu, Nanjing, China). The sample size for animal studies was determined based on a survey of data from published research or preliminary studies,^15^ and no mice were excluded from the statistical analysis. These mice were housed in specific pathogen‐free (SPF) units at the Animal Center of Shenzhen People's Hospital. They were maintained under a 12‐hour light cycle from 8 am to 8 pm, at a temperature of 23 ± 1°C and humidity of 60–70%. The mice were provided with a standard rodent diet and had free access to water in plastic bottles. Prior to the experiments, the mice were allowed to acclimate to their housing environment for a minimum of 7 days. Up to five mice were housed per plastic cage, which contained corn cob bedding material. The mice were treated in a blinded manner, and randomization was performed before administering the treatments. At the end of the experiment, all mice were anaesthetised and euthanized in a CO_2_ chamber. Blood or muscle samples were then collected.

### Dexamethasone‐induced muscle atrophy mouse model

Dexamethasone (DEX)‐induced muscle atrophy models were developed following previously described procedures.^16^ Briefly, 8 week‐old male wild‐type (WT) and KLF13 KO mice were randomly assigned to four groups (*n* = 6 in each group). The control group received a PEG‐solution (10 mL/kg body weight, 30% in 0.9% saline), while the DEX group was administered DEX (25 mg/kg dissolved in PEG 400 solution) alone or with clofoctol (10 mg/kg clofoctol i.p. daily) intervention via intraperitoneal injection for 10 days. Mouse body weight was recorded daily, and grip strength was measured three times using a grip strength test‐meter. Twenty‐four hours after the last intraperitoneal injection, the mice were euthanized. The gastrocnemius (Gas), tibialis anterior (TA), extensor digitorum longus (EDL) and soleus (SOL) muscles were isolated for further analysis.

### Cisplatin‐induced muscle atrophy

Using saline (vehicle) as a control, C57BL/6 mice and KLF13‐KO mice were treated with cisplatin at a dose of 3 mg/kg once daily for five consecutive days via intraperitoneal administration.

### Diabetes mice

C57BL/6J mice (8 ± 0.5 weeks old and weighing 24 ± 1 g bodyweight) were randomly assigned to two groups: a control group and an HFD/STZ group, with each group consisting of six animals. The mice in the HFD/STZ group were fed a high‐fat diet (Cat. No. MD12033; Research Diets, New Brunswick, NJ, USA) for 4 weeks. They were then fasted for 12 h and received daily intraperitoneal injections of 50 mg/kg body weight of streptozotocin (STZ) (Sigma‐Aldrich, S0130; dissolved in 50 mM citric acid buffer, pH 4.5) for five consecutive days. After STZ administration, the diabetic mice were maintained on the high‐fat diet for 12 weeks. The mice in the control group received the same volume of citric acid buffer and were fed a standard normal chow diet (with 10% of calories from fat). Fasting blood glucose levels were measured using a glucometer (ACCU‐CHEK) from blood samples collected from the tail vein 9 days after the first STZ injection. The mouse model of HFD/STZ was considered successfully established if there were two consecutive fasting blood glucose levels higher than 16.7 mM. To overexpress KLF13, control mice and HFD/STZ mice were administered in situ Gas with AAV9‐Ctrl (control group) or AAV9‐Klf13 [Klf13 overexpression group, 0.5–1.5 × 10^11^ viral genomes [vg]/mL in 50 μl saline (0.15 mol/L NaCl); GeneChem Company, Shanghai, China] once when they were 13 weeks old. The mice were then divided into the control group, control + K13OE group, HFD/STZ group and HFD/STZ + K13OE group, with six mice included in each group for the indicated experiments.

### Quantitative real‐time PCR

Total RNAs were extracted by Trizol (Invitrogen) and then dissolved in an appropriate amount of RNase water. cDNA was obtained by a reverse transcription kit purchased from TransGen Biotech (Beijing, China). qPCR was performed using the ABI StepOnePlusTM Real‐time PCR system (Applied Biosystems) with specific primers (*Table* *S2*). The relative mRNA levels of target genes were analysed using the 2^−ΔΔCT^ method.^17^, ^18^ Tubulin was used as a housekeeping gene for analysis.

### Study approval

All animal care and experimental protocols for in vivo studies conformed to the Guide for the Care and Use of Laboratory Animals, published by the National Institutes of Health (NIH; NIH publication no.: 85–23, revised 1996), was approved by the Animal Care Committees of Shenzhen People's Hospital (No. AUP‐230905‐LZ‐0481‐01), and were performed in compliance with the ARRIVE guidelines.

### Quantification and statistical analysis

All data were generated from at least three independent experiments. Each value was presented as the mean ± *SD*. All raw data were initially subjected to a normal distribution and analysis by one‐sample Kolmogorov–Smirnov (K–S) nonparametric test using SPSS 22.0 software. For animal and cellular experiments, a two‐tailed unpaired Student's *t*‐test was performed to compare the two groups. One‐way ANOVA followed by the Bonferroni's post‐hoc test was used to compare more than two groups. To avoid bias, all statistical analyses were performed blindly. Statistical significance was indicated at *P < 0.05, **P < 0.01 and ***P < 0.001.

## Results

### KLF13 is down‐regulated in mouse models of muscle atrophy

**Figure 1:**
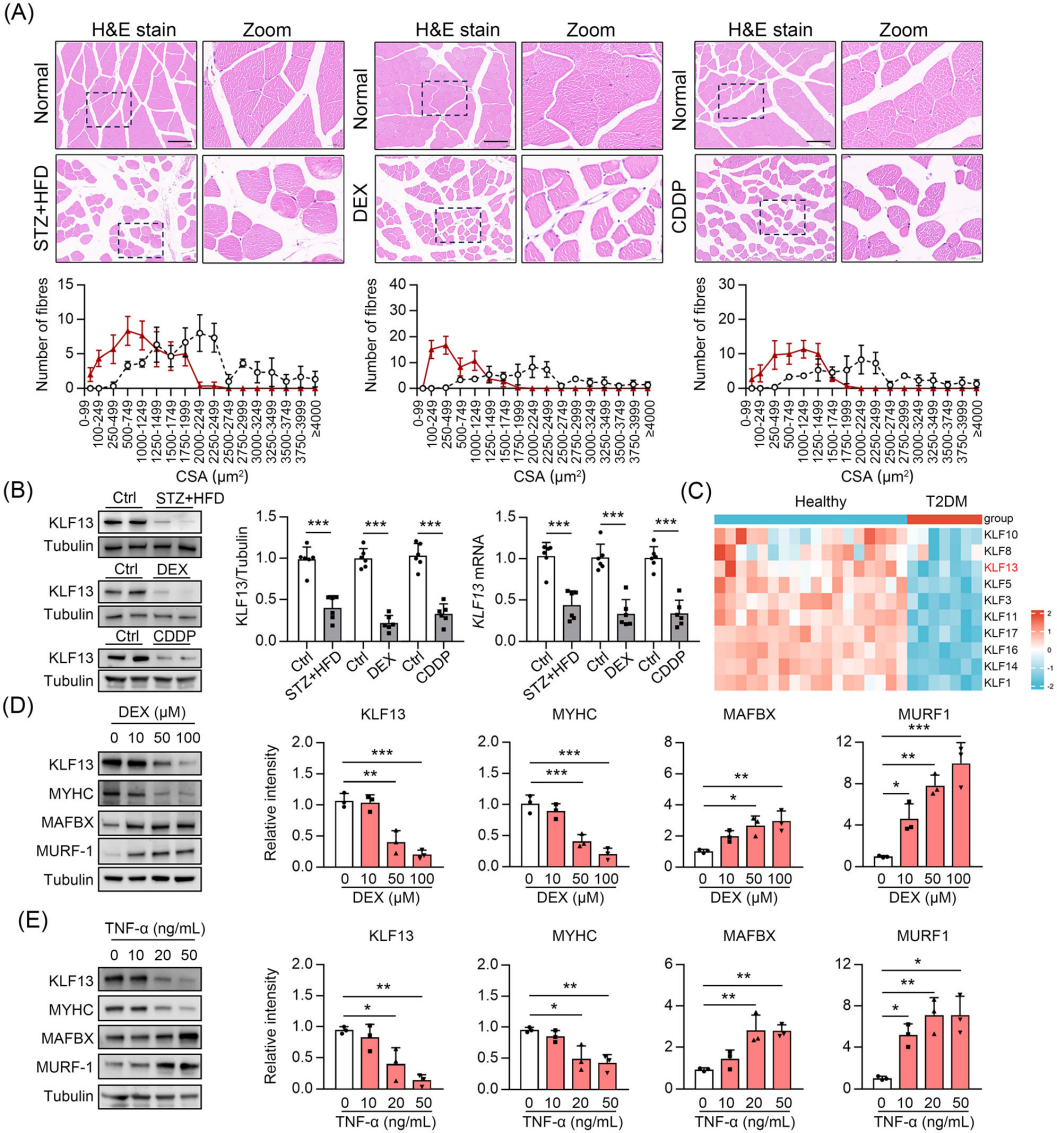
KLF13 was reduced in human and mouse models of muscle atrophy. (*A*) Representative image of haematoxylin and eosin (H&E) staining of cross‐sectioned myofibres isolated from mouse gastrocnemius maximus (Gas) muscles. The images were captured using a microscope with a × 20 objective. (Below) Cross‐sectional area (CSA) of Gas muscles from mice, *n* = 3 (scale bar = 100 μM). (*B*) Immunoblot analysis of KLF13 proteins (left) and qPCR analysis of *KLF13* mRNA (right) in gas muscles from diabetic mice (STZ + HFD), wild type (WT) mice treated with DEX or CDDP. (Middle) Quantification represents the levels of the indicated protein normalized to tubulin, *n* = 6. (*C*) RNA sequencing analysis (GSE156249) of skeletal muscles from patients with diabetes and healthy control. (*D*, *E*) Immunoblot analysis of KLF13, MYHC, MAFBX and MURF‐1 in C2C12 cells treated with DEX (*D*) or TNFα (*E*). (Right) Quantification represents the levels of the indicated protein normalized to tubulin, *n* = 3. Data are expressed as mean ± *SD*. In subpart (*B*), *P < 0.05, **P < 0.01, ***P < 0.001, by unpaired Student's *t* test. In subparts (*D*, *E*): *P < 0.05, **P < 0.01, ***P < 0.001, by one‐way ANOVA with Bonferroni correction.

First, we investigated changes in endogenous KLF13 expression in several muscle atrophy mouse models to determine if *KLF13* is directly involved in the pathogenesis. We found that the Gas and TA muscles of diabetic mice (STZ + HFD), WT mice treated with DEX or CDDP had lower KLF13 protein levels than their respective control groups (*Figures* *1* *A,B* and *S1A,B*). The analysis of publicly available transcriptomics data (dataset GEO: GSE156249) further demonstrates that KLF13 expression is downregulated in skeletal muscle from patients with diabetes compared with healthy controls (*Figure* *1* *C*). Two muscle‐specific E3 ubiquitin ligases, muscle atrophy F‐box (MAFBX) and muscle RING‐finger protein 1 (MURF‐1), are upregulated with atrophy resulting from cachexia and disuse. In addition, MURF‐1 causes the breakdown of myosin heavy chain (MYHC) and other thick filament components of the sarcomere during atrophy.^19^, ^20^ We also found decreased MYHC and increased MAFBX and MURF‐1 expression in C2C12 cells treated with DEX or tumour necrosis factor‐alpha (TNF‐α) (*Figure* *1* *D,E*). Furthermore, DEX or TNF‐α‐stimulation dose‐dependently decreased KLF13 protein levels (*Figure* *1* *D,E*). These results suggest that KLF13 is downregulated in skeletal muscles during muscle atrophy.

### KLF13 ablation aggravates muscle loss in glucocorticoids or *cis*‐platinum‐induced muscle atrophy in mice

**Figure 2:**
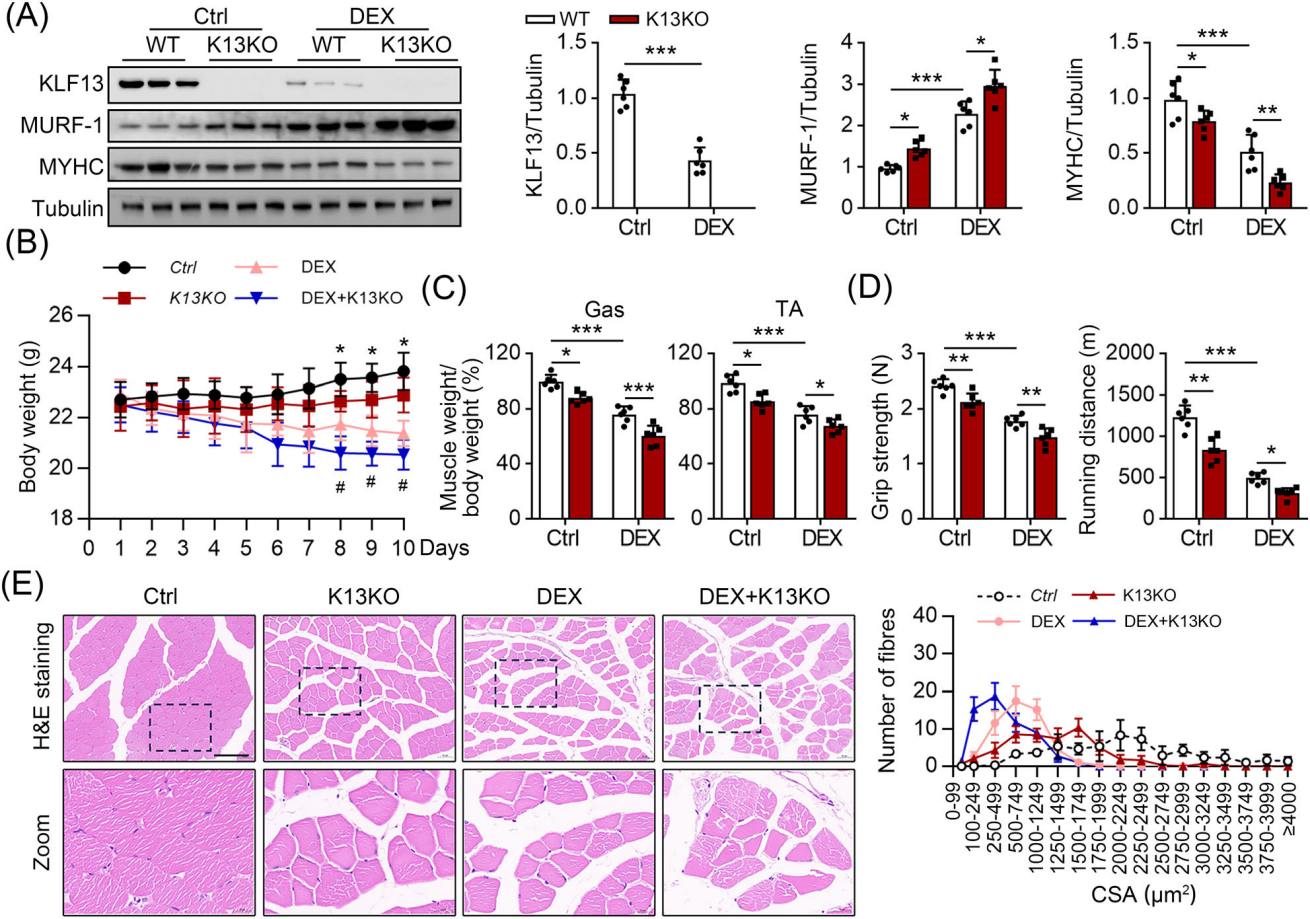
Knockout of KLF13 exacerbated glucocorticoids‐induced muscle atrophy in mice. We established a DEX‐induced muscle atrophy model in both WT and K13KO mice. (*A*) Immunoblots analysis of KLF13, MURF‐1 and MYHC using Gas muscle. (Right) Quantification represents the levels of the indicated protein normalized to tubulin, *n* = 6. (*B*) Total body weight of mice, *n* = 6. (*C*) The ratio of Gas muscle and TA muscle weight to body weight, *n* = 6. (*D*) Grip strength test and exhaustive running distance, *n* = 6. (*E*) Representative images of myofibre cross‐sections were obtained through H&E staining. (right) the cross‐sectional area (CSA) of gas muscles from mice, *n* = 6 (scale bar = 100 μM). Data are expressed as mean ± *SD*. In subparts (*A*, *C*, *D*): *P < 0.05, **P < 0.01, ***P < 0.001, by one‐way ANOVA with Bonferroni correction. In subpart (*B*): *P < 0.05, **P < 0.01, ***P < 0.001, by two‐way ANOVA with Bonferroni correction.

We established a negative correlation between KLF13 expression and muscle atrophy in vivo; thus, we used a DEX‐induced in vivo model to investigate how KLF13 promotes muscle atrophy.^21^ First, we generated *KLF13* knockout (K13KO) mice (*Figure* *2* *A*), finding that the K13KO mice had a lower body weight than the WT mice (*Figure* *2* *B*), but the knockout did not affect the heart weight/tibia length ratio and daily food intake level (data not shown). However, the MURF‐1 increased, while the MYHC protein levels decreased, in the Gas of K13KO mice compared with the WT mice (*Figure* *2* *A*). Similarly, the K13KO mice had lower Gas and TA muscle weights than the WT mice (*Figure* *2* *C*), as well as decreased grip strength and a shorter exhaustive running distance (*Figure* *2* *D*). Next, basic histological evaluations of the glycolytic Gas muscles revealed that both the K13KO and control mice had a normal muscle structure (*Figure* *2* *E*). However, the Gas of K13KO mice had more small myofibres and a left‐shifted distribution profile compared with the control mice (*Figure* *2* *E*). Next, we examined the function of KLF13 in muscle atrophy using a DEX‐induced muscle atrophy mouse model. Aligning with in vitro results, the KLF13 protein level in the Gas was lower in the DEX treatment group than in the control group (*Figure* *2* *A*). In addition, DEX treatment significantly reduced the body weight, Gas and TA muscle mass, grip strength and exhaustive running distance, and the DEX‐induced mice had more small myofibres compared with the control mice (*Figure* *2* *B–E*). K13KO augmented the effects of DEX on muscle atrophy, evidenced by higher MURF‐1 and lower MYHC protein levels, decreased Gas and TA muscle masses and grip strength, a shorter exhaustive running distance and more small‐sized myofibres compared with the WT mice treated with DEX (*Figure* *2* *B–E*). Similar results were observed for mice with cisplatin‐induced skeletal muscle atrophy (*Figure* *S2A–E*). These data suggest that K13KO causes muscle loss and weakness.

### Overexpression of KLF13 protects against diabetes or glucocorticoids ‐induced muscle atrophy mice

**Figure 3:**
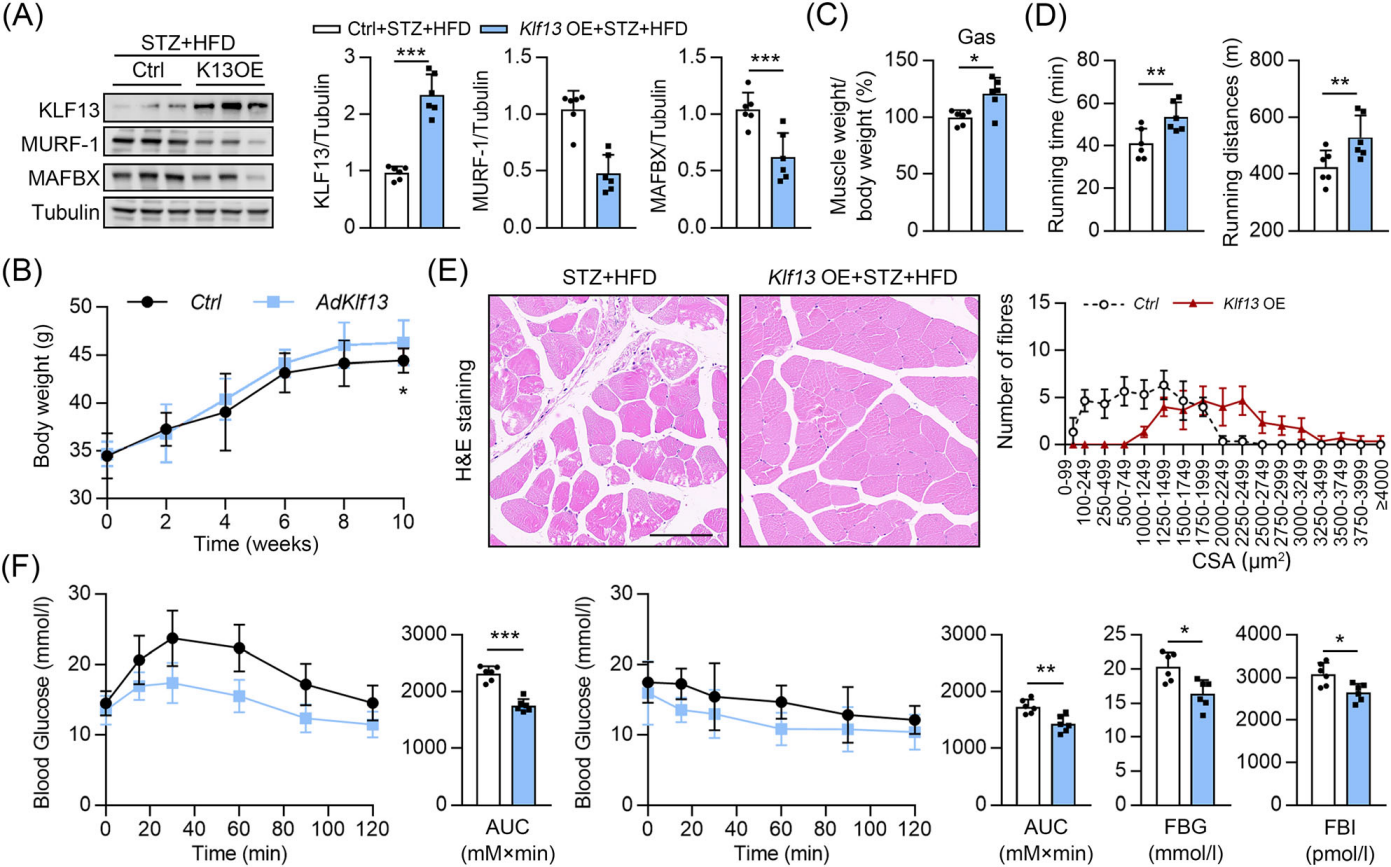
KLF13 overexpression alleviated diabetes‐induced muscle atrophy in mice. These analyses were performed in both wild‐type (WT) and KLF13 overexpressing (K13OE) mice using a diabetes‐induced model with STZ and a HFD. (*A*) Immunoblots analysis of KLF13, MAFBX and MURF‐1 in Gas muscle. (Right) Quantification represents the levels of the indicated protein normalized to tubulin, *n* = 6. (*B*) Total body weight, *n* = 6. (*C*) The ratio of Gas muscle weight to total body weight, *n* = 6. (*D*) Grip strength test and exhaustive running distance, *n* = 6. (*E*) Representative image of H&E staining of myofibre cross‐section from Ctrl or *AAV‐Klf13* injected into Gas muscles of diabetes mice. (Right) Cross‐sectional area (CSA) of Gas muscles from mice, *n* = 6 (scale bar = 100 μM). (*F*) Blood glucose during ITT and GTT, as well as fasting blood glucose (FBG) and fasting blood insulin (FBI), *n* = 6. Data are expressed as mean ± *SD*. In subparts (*A*, *C*, *D*, *F*): *P < 0.05, **P < 0.01, ***P < 0.001, by unpaired Student's *t* test. In subpart (*B*): *P < 0.05, by two‐way ANOVA with Bonferroni correction.

Next, KLF13 or control adeno‐associated virus 9 (AAV9) vectors were injected in situ into the Gas of diabetic mice (STZ + HFD; spontaneous diabetes mouse model) to evaluate the effects of *KLF13* overexpression on muscle atrophy. Compared with the controls, *KLF13* expression significantly increased in the Gas muscles after AAV9 transfection (*Figure* *3* *A*) without influencing the heart weight/tibia length ratio or the daily food intake level (data not shown). *KLF13* overexpression slightly increased the body weight (*Figure* *3* *B*). Consistently, the Gas muscle mass significantly increased, and the MURF‐1 and MAFBX protein levels in the Gas muscles significantly decreased after *KLF13* overexpression (*Figure* *3* *A,C*). Consistently, *KLF13* overexpression enhanced skeletal muscle performance and endurance in the diabetic mice (assessed by exhaustive running time and distance) (*Figure* *3* *D*). Finally, the diabetic mice had a normal muscle structure but fewer small myofibres in the Gas muscles after KLF13 overexpression (*Figure* *3* *E*). In prediabetic and healthy individuals, skeletal muscle clears over 80% of the glucose.^22^ We found that KLF13 overexpression in the skeletal muscles enhanced insulin sensitivity in diabetic mice, evidenced by lower fasting serum glucose and insulin levels (*Figure* *3* *F*) and lower glucose levels in the insulin and glucose tolerance tests (*Figure* *3* *F*). Similar results were observed for mice with DEX‐induced skeletal muscle atrophy (*Figure* *3A–E*). Collectively, the overexpression of KLF13 in skeletal muscle mitigated muscle loss in both diabetic mice and mice with DEX‐induced muscle atrophy.

### KLF13 represses myofibre atrophy through inhibiting Dll4 expression

**Figure 4:**
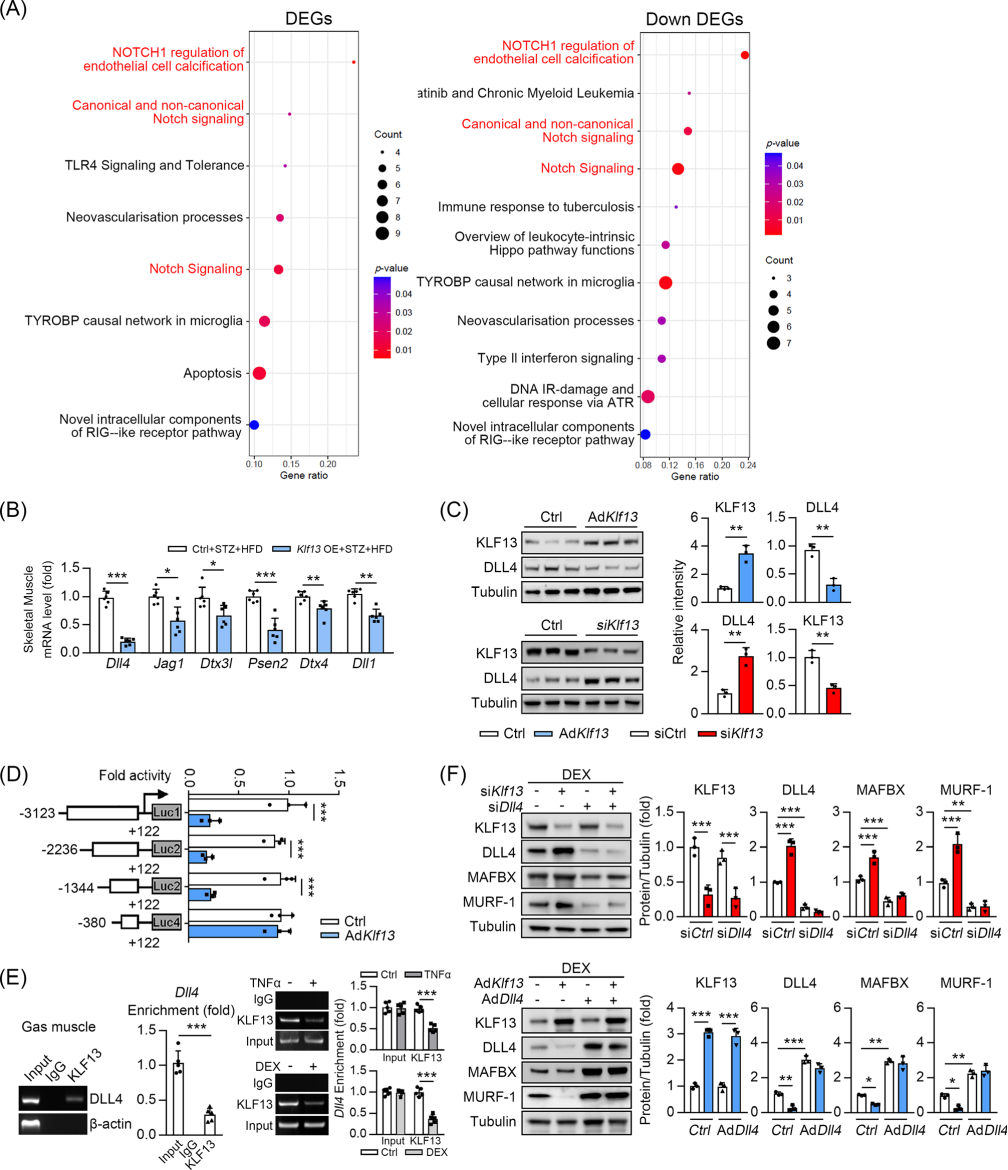
KLF13 transcriptional repressed the DLL4. (*A*) The ingenuity pathway analysis (IPA) of RNA‐seq in Gas muscle of WT and KLF13 overexpression mice. (*B*) qPCR analysis was used to detect mRNA levels of Dll4, Jag1, Dtx3l, Psen2, Dtx4 and Dll1 in TA muscle from WT and KLF13OE mice, *n* = 6. (*C*) C2C12 myotubes were transfected with the indicated adenovirus (Ad‐Klf13) /siKlf13 and Ctrl in the presence of DEX 50 μM for 24 h. Immunoblots analysis of the expression of Dll4. (Right) Quantification represents the levels of the indicated protein normalized to tubulin, *n* = 3. (*D*) Luciferase reporter assays showing the activity of different truncation of Dll4 promoter fragments in HEK293T cells, *n* = 3. (*E*) In kidney from mice, DNA fragments containing KLF13 binding site on the *Dll4* promoter were immunoprecipitated with anti‐KLF13 and then PCR amplified (left panel). TNFα or DEX were treated with C2C12 myotubes as indicated and then DNA fragments containing KLF13 binding site on the *Dll4* promoter were immunoprecipitated with anti‐KLF13 and then PCR amplified (right panel). (*F*) Immunoblot analysis of indicated proteins in C2C12 myotubes transfected with siRNA Klf13 (si*Klf13*) and Dll4 (si*Dll4*) (or Ad*Klf13* and Ad*Dll4*) simultaneously or individually in the presence of dexamethasone (DEX) 50 μM for 24 h. (Right) Quantification represents the levels of the indicated protein normalized to tubulin, *n* = 3. Data are expressed as mean ± *SD*. In subparts (*B*, *C*): *P < 0.05, **P < 0.01, CDDP+K13KO vs CDDP, by unpaired Student's *t* test. In subparts (*D*, *F*): *P < 0.05, **P < 0.01, ***P < 0.001, by one‐way ANOVA with Bonferroni correction.

We identified 887 differentially expressed genes in the Gas of mice between the AAV9‐KLF13 and AAV9‐control groups; 230 were significantly upregulated, and 657 were significantly downregulated (*P* < 0.05, *Table S1*). An ingenuity pathway analysis revealed that ‘NOTCH1 regulation of endothelial cell calcification’, ‘canonical and non‐canonical Notch Signaling’ and ‘Notch signalling’ were the top pathways affected by *KLF13* overexpression (*Figure* *4* *A*). KLF13 is a well‐known transcription repressor, and *KLF13* overexpression repressed genes related to ‘Notch signalling’ [*Dll4*, jagged canonical notch ligand 1 (i.e., *Jag1*), deltex E3 ubiquitin ligase 3L (i.e., *Dtx3l*), presenilin 2 (i.e., *Psen2*) and deltex E3 ubiquitin ligase 4 (i.e., *Dtx4*)] in the skeletal muscle of diabetic mice (STZ + HFD) compared with the control mice (*Figure* *4* *B*). Using the JASPAR database (https://jaspar.genereg.net/), we found that of these genes, only *Dll4* (a Notch ligand) had a KLF response element, suggesting that *Dll4* could be a direct target of KLF13. Next, we confirmed that DLL4 protein levels were downregulated in C2C12 cells after *KLF13* overexpression (*Figure* *4* *C*). Consistency, *KLF13* knockdown upregulated *DLL4* mRNA and protein levels in C2C12 cells (*Figure* *4* *C*). Next, we performed several assays using various luciferase reporter constructs containing the human *DLL4* gene promoter to determine the molecular basis for DLL4 downregulation by KLF13. *KLF13* overexpression restrained −3123Luc, −2236Luc and −1344Luc transcription but not −380Luc transcription in HEK293T cells (*Figure* *4* *D*), suggesting that this region, spanning from −1344 bp to −380 bp, is required for the transcriptional action of DLL4 by KLF13. In addition, two potential KLF binding sites were identified in the −1344 to −380 region of the DLL4 promoter via bioinformatic analyses (i.e., JASPER). Next, chromatin immunoprecipitation assays (ChIP) were performed to investigate whether endogenous KLF13 protein could directly bind to this region of the *DLL4* promoter in vivo (*Figure* *4* *E*, left panel). The *Dll4* promoter fragment containing KLF13 binding site was amplified from precipitates derived from the kidneys of mice using an anti‐KLF13 antibody (*Figure* *4* *E*, left panel). Furthermore, we found that TNFα or DEX treatment restrained the binding of KLF13 in the promoter of *Dll4* in vitro (*Figure* *4* *E*, right panel). *Dll4* deficiency repressed the regulation of atrogenes (MURF‐1 and MAFBX) by *Klf13* siRNA (si*Klf13*) in C2C12 cells (*Figure* *4* *F*). Consistently, *KLF13* overexpression did not affect MURF‐1 or MAFBX regulation when *DLL4* was overexpressed (*Figure* *4* *F*). Furthermore, in mice with DEX‐ or cisplatin‐induced muscle atrophy, *KLF13* knockout upregulated DLL4 mRNA and protein expression in the Gas muscles (*Figure* *4A,B*). *KLF13* overexpression also inhibited DLL4 expression in the Gas muscles of diabetic mice (*Figure* *S4C*). These results demonstrated that KLF13 transcription inhibits DLL4 expression by binding to its promoter region.

### KLF13 prevents glucocorticoids‐induced muscle atrophy

**Figure 5:**
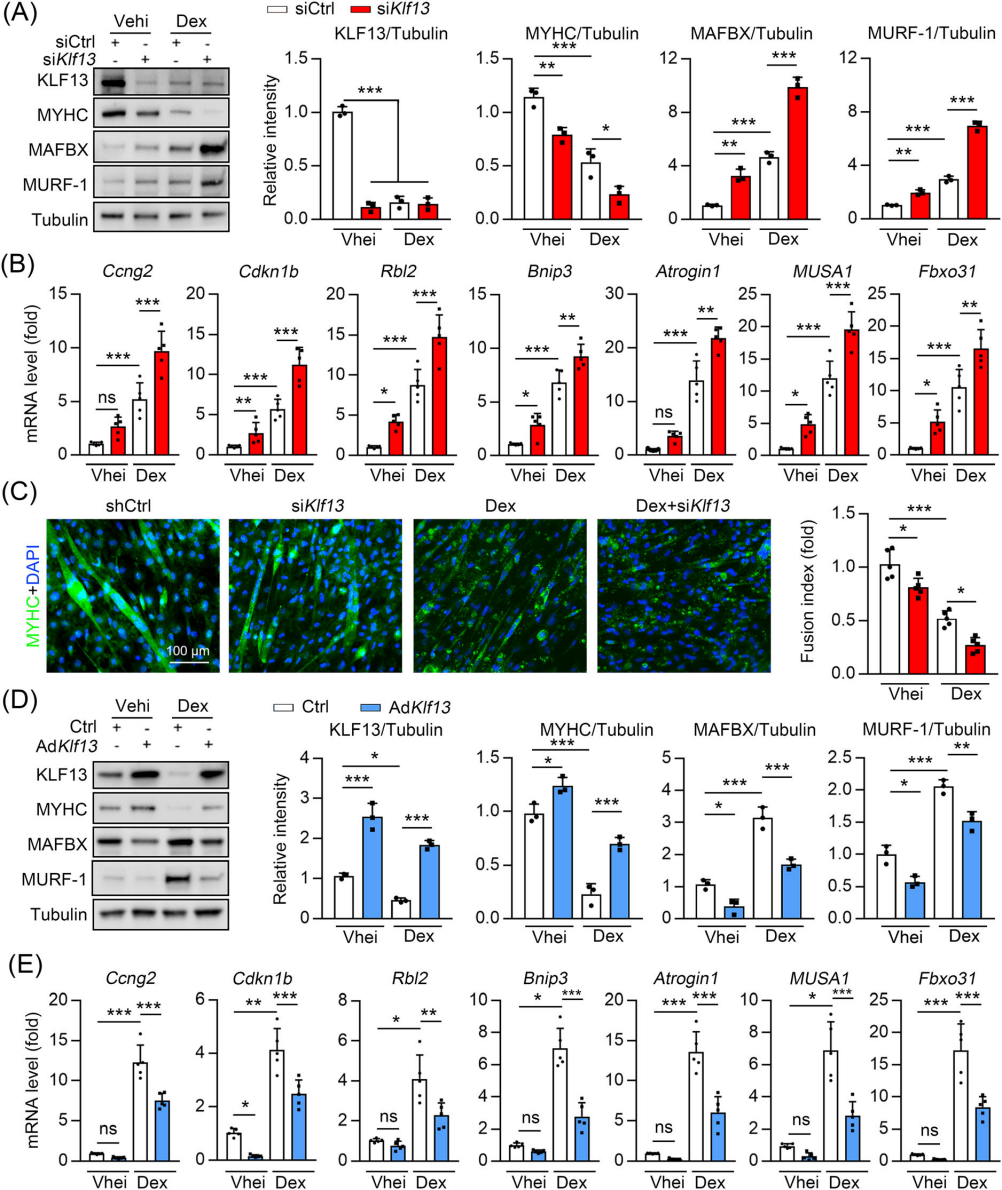
KLF13 prevents glucocorticoids‐induced muscle atrophy in vitro. (*A*) Immunoblot analysis of KLF13, MYHC, MAFBX and MURF‐1 in C2C12 myotubes transfected with control or *Klf13* siRNA (si*Klf13*) in the presence or absence of DEX at 50 μM for 24 h. (Right) Quantification represents the levels of the indicated protein normalized to tubulin, *n* = 3. (*B*) qPCR analysis of *Ccng2*, *Cdkn1b*, *Rbl2*, *Bnip3*, *Atrogin1*, *MUSA1* and *Fbxo31* in C2C12 myotubes transfected with control or *Klf13* siRNA and treated with or without DEX at 50 μM for 24 h, *n* = 5. (*C*) MYHC immunofluorescence of C2C12 myotubes transfected with control or *Klf13* siRNA and treated with or without DEX at 50 μM for 24 h. (Right) Quantification represents the levels of fusion index, *n* = 5. (*D*) Immunoblots analysis of KLF13, MYHC, MAFBX and MURF‐1 in C2C12 myotubes infected with adenovirus expressing KLF13 (Ad*KLf13*) or GFP (Ctrl). Myotubes were culture for 24 h in the presence or absence of DEX at 50 μM. (Right) Quantification represents the levels of the indicated protein normalized to tubulin, *n* = 3. (*E*) qPCR analysis of *Ccng2*, *Cdkn1b*, *Rbl2*, *Bnip3*, *Atrogin1*, *MUSA1* and *Fbxo31* in C2C12 myotubes infected with Ad*KLf13* or Ctrl and treated with or without DEX at 50 μM for 24 h, *n* = 5. Data are expressed as mean ± *SD*. In subparts (*A–E*): *P < 0.05, **P < 0.01, ***P < 0.001, by one‐way ANOVA with Bonferroni correction.

Next, we created an in vitro DEX‐induced model by challenging cells with synthetic DEX to investigate the role of KLF13 in the development of muscle atrophy.^21^ We also performed siRNA‐mediated targeted KLF13 knockdown (si*Klf13*) and selected the optimal DEX‐stimulatory dose (50 μM) to mimic muscle atrophy in C2C12 myotubes to further explore if *KLF13* inhibition could aggravate DEX‐induced muscle atrophy phenotype. Based on the protein expression, we confirmed *KLF13* knockdown (*Figure* *5* *A*). MAFBX and MURF‐1 expressions were also upregulated in the DEX‐treated cells, which was further enhanced by si*Klf13* transfection (*Figure* *5* *A*). Consistently, the DEX‐triggered decrease of MYHC protein levels was enhanced in the *KLF13* depleted cells (*Figure* *5* *A*). Reports indicate that Notch2 (the major receptor of DLL4) is an upstream regulator of forkhead box subgroup O (FoxO) signalling, which is central to induce muscle atrophy.^4^ Consistently, we found that DEX increased the expression of FoxO signalling pathway‐related genes, including cyclin G2 (i.e., *Ccng2*), cyclin‐dependent kinase inhibitor 1B (i.e., *Cdkn1b*), retinoblastoma‐like protein 2 (i.e., *Rbl2*) and Bcl‐2 interacting protein 3 (i.e., *Bnip3*), and that these changes were further enhanced without *KLF13* (*Figure* *5* *B*). Furthermore, the mRNA levels of ubiquitin ligases [e.g., Atrogin‐1, muscle ubiquitin ligase of SCF complex in atrophy‐1 (i.e., *MUSA1*) and F‐box protein 31 (i.e., *Fbxo31*)], which are known to induce muscle atrophy,^23^, ^24^ were increased in the DEX‐treated cells, and *KLF13* knockout augmented these effects (*Figure* *5* *B*). Immunofluorescence staining demonstrated that *KLF13* knockdown amplified the DEX‐triggered decrease in MYHC staining, cell fusion and multinucleated myotube formation (*Figure* *5* *C*). We also observed that adenovirus‐mediated *KLF13* overexpression repressed MAFBX and MURF‐1 upregulation and MYHC downregulation upon DEX stimulation (*Figure* *5* *D*). *KLF13* overexpression also suppressed the DEX‐induced expression of FoxO target genes and atrogenes (*Figure* *5* *F*). Collectively, these results suggest that KLF13 prevents DEX‐induced myotube atrophy.

### Myod1 transcript regulates KLF13

**Figure 6:**
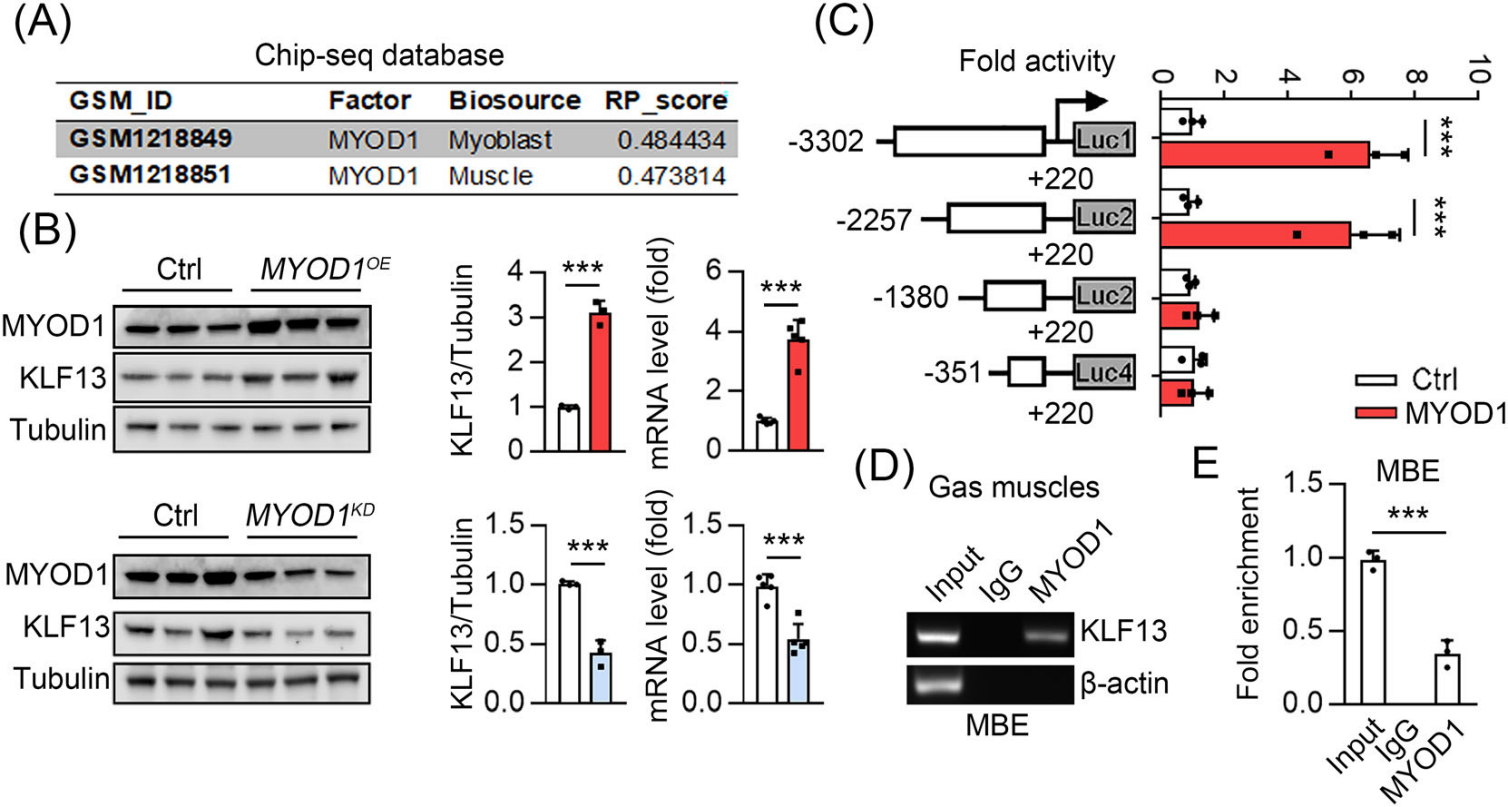
MYOD1 transcriptional activated the KLF13. (*A*) ChIP‐seq data analysis showed MYOD1 binding to the promoter of KLF13 in muscle and myoblast. (B) Protein level of KLF13 in C2C12 cells infected with adenovirus expressing MYOD1 (MYOD1^OE^) or MYOD1 shRNA (MYOD1^KD^). (Right) Quantification represents the levels of the indicated protein normalized to tubulin (*n* = 3) and qPCR analysis was used to detect mRNA levels of KLF13 (*n* = 5). (C) Luciferase reporter assays showing the activity of different truncation of KLF13 promoter fragments in HEK293T cells, *n* = 3. (*D*, *E*) ChIP‐qPCR assays of the binding of MYOD1 to MBE regions of the *Klf13* promoter in the Gas muscle from mice, *n* = 3. Data are expressed as mean ± *SD*. In subpart (*B*): *P < 0.05, **P < 0.01, CDDP+K13KO versus CDDP, by unpaired Student's *t* test. In subparts (*C*–*E*): *P < 0.05, **P < 0.01, ***P < 0.001, by one‐way ANOVA with Bonferroni correction.

We found decreased *KLF13* transcript levels in the Gas muscles of mice with muscle atrophy (*Figure* *1* *A*, *B*). Therefore, we reanalysed the Chip‐seq data obtained from the Cistrome Data Browser (http://cistrome.org/db/#/), finding that MYOD1 binds the KLF13 promoter in muscle and myoblasts (*Figure* *6* *A*). MYOD1 (or simply MYOD) plays pivotal roles in muscle differentiation, development and regeneration. Studies have consistently reported downregulated MYOD1 expression during skeletal muscle atrophy, leading to decreased functionality.^25^, ^26^ Hence, we first determined whether MYOD1 regulates KLF13 expression in C2C12 cells, finding that *MYOD1* overexpression increased and *MYOD1* knockdown repressed the KLF13 mRNA and protein levels (*Figure* *6* *B*). Thus, we performed assays with several luciferase reporter constructs containing the human *KLF13* gene promoter, finding that *MYOD1* overexpression enhanced the transcription of −3302Lucand −2257Luc, but not −1380Lucor –351Luc in HEK293T cells (*Figure* *6* *C*). Therefore, the MYOD1 binding site within the *KLF13* promoter was between −2257 and −1380 bp. Only one putative MYOD1 binding motif sequence (MBE) was predicted in the *KLF13* promoter region (JASPAR analysis), and ChIP‐quantitative polymerase chain reaction assays further demonstrated the binding of MYOD1 to the MBE (−1501 to −1321 bp) of the *KLF13* promoter (*Figure* *6* *D,E*). Collectively, these results demonstrated that MYOD1 transcriptionally activates KLF13 expression by binding to its promoter region.

### Fbxw7 controls proteasome‐mediated degradation of KLF13

**Figure 7:**
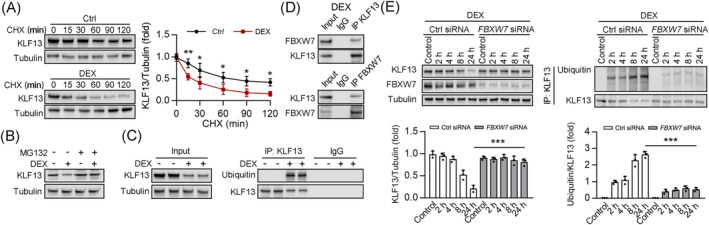
FBXW7 mediated the ubiquitination and degradation of KLF13 *in vitro*. (*A*) C2C12 cells treated with CHX in the presence or absence ofDEX 50 μM as indicated in figure, and the western blot analysis of KLF13 and tubulin were shown in the left panel, and the quantitative results are shown in the right panel, *n* = 3. (*B*) C2C12 myotubes were treated with or without DEX 50 μM for 24 h, treated MG132 10 μM for 6 h. Immunoblot analysis of KLF13 and tubulin as indicated. (*C*) C2C12 myotubes were treated with or without DEX 50 μM for 24 h. Co‐immunoprecipitation (co‐IP) using KLF13 antibody and immunoblot analysis of input KLF13 (left) and ubiquitin (right). (*D*) HEK293T cells were treated with DEX 50 μM for 24 h. co‐immunoprecipitation (co‐IP) using KLF13 or FBXW7 and immunoblot analysis of indicated proteins. (*E*) HEK293T cells transfected with ctrl siRNA or *FBXW7* siRNA for 24 h, and treated with DEX 50 μM for different hours as indicated. Western blot analysis of KLF13 and tubulin, with the quantitative results shown in the below panel, *n* = 3. HEK293T cells lysates were subjected to Co‐IP with anti‐KLF13 antibody, followed by western blotting using ubiquitin antibodies. The quantitative results are shown in the below panel, *n* = 3. Data are expressed as mean ± *SD*. In subpart (*A*): *P < 0.05, **P < 0.01, by two‐way ANOVA with Bonferroni correction. In subpart (*E*): ***P < 0.001, by one‐way ANOVA with Bonferroni correction.

Glycogen synthase kinase 3β (GSK3β)‐mediated KLF13 phosphorylation triggers the KLF13 ubiquitination by the E3 ligase, Fbxw7γ, resulting in KLF13 protein degradation.^15^, ^27^ Fbxw7γ is largely expressed in heart and skeletal muscle,^28^ and DEX treatment increases GSK3β activity.^29^ Intriguingly, we found that DEX stimulation shortened the half‐life of KLF13 (*Figure* *7* *A*) and that MG132 (a proteasome inhibitor) blocked DEX‐induced KLF13 degradation (*Figure* *7* *B*), but NH_4_Cl (a lysosome inhibitor) and 3‐MA (an autophagosome inhibitor) did not (data not shown). Furthermore, DEX stimulation induced KLF13 ubiquitination (*Figure* *7* *C*). Consistently, KLF13 and FBXW7 interacted under DEX stimulation (*Figure* *7* *D*). Importantly, *FBXW7* knockdown (via siRNA) decreased the DEX‐mediated KLF13 degradation and ubiquitination compared with control‐transfected cells (*Figure* *7* *E*). Therefore, DEX triggers the KLF13 ubiquitination by the E3 ligase, Fbxw7γ, resulting in KLF13 protein degradation.

### Clofoctol alleviates muscle atrophy induced by dexamethasone

**Figure 8:**
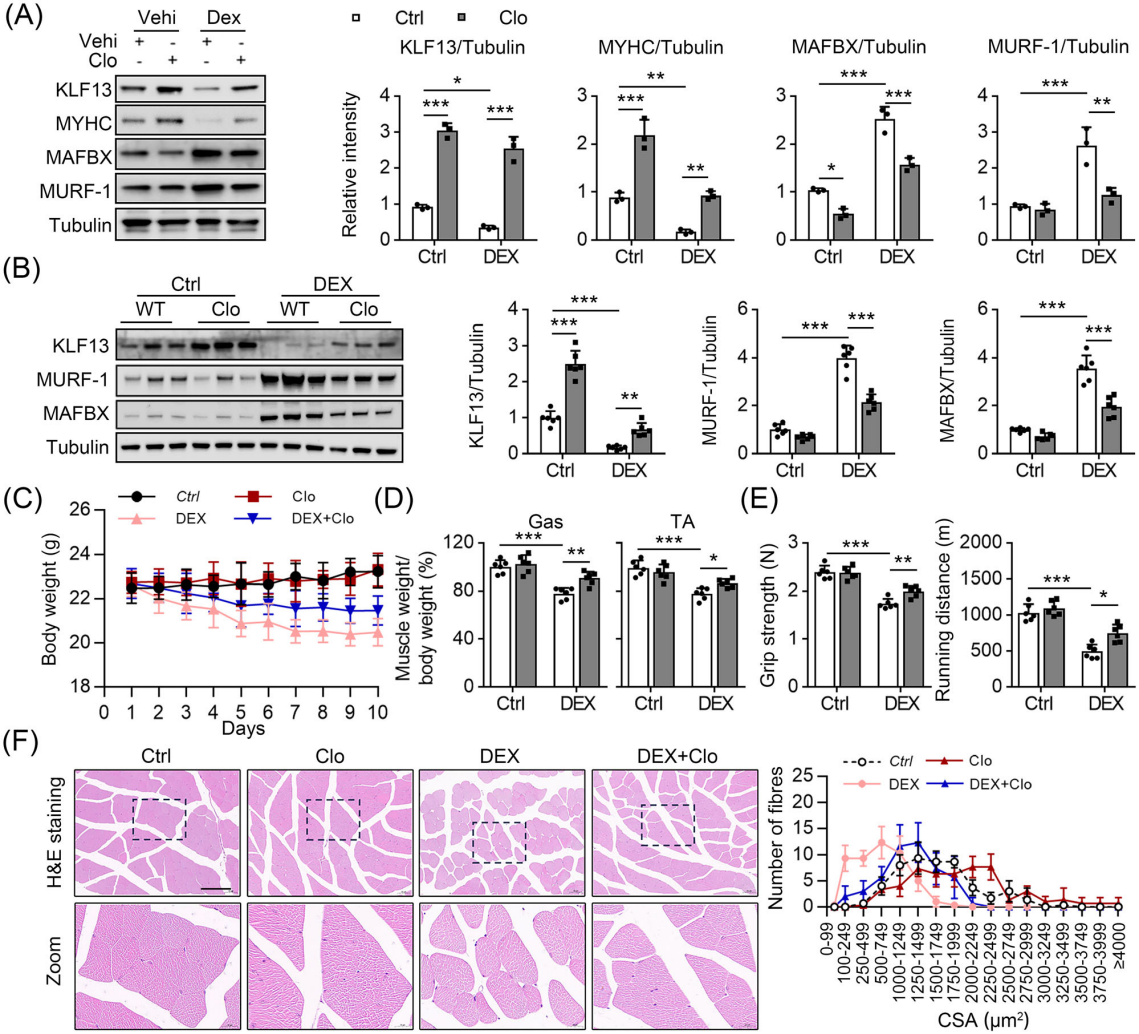
Clofoctol intervention prevents muscle loss in glucocorticoids‐induced muscle atrophy in mice. (*A*) C2C12 cells treated with Clo in the presence or absence of DEX 50 μM as indicated in figure, and the western blot analysis of KLF13, MYHC, MAFBX, MURF‐1 and tubulin were shown in the left panel, and the quantitative results are shown in the right panel, *n* = 3. (*B*) We established a DEX‐induced muscle atrophy model in both WT and Clo‐treated mice. Immunoblots analysis of indicated proteins using Gas muscle. (Right) Quantification represents the levels of the indicated protein normalized to tubulin, *n* = 6. (*C*) Total body weight, *n* = 6. (*D*) The ratio of Gas or TA muscle weight to body weight, *n* = 6. (E) Grip strength test and exhaustive running distance, *n* = 6. (F) Representative images of myofibre cross‐sections were obtained through H&E staining. (Right) Cross‐sectional area (CSA) of Gas muscles from mice, *n* = 6 (scale bar = 100 μM). Data are expressed as mean ± *SD*. In subparts (*A*, *B*, *D*, *E*): ***P < 0.001, by one‐way ANOVA with Bonferroni correction. In subpart (*C*): *P < 0.05, **P < 0.01, by two‐way ANOVA with Bonferroni correction.

Clofoctol (Clo) induces KLF13^30^; hence, we first determined whether Clo could upregulate the mRNA and protein level of KLF13 in C2C12 cells. As expected, KLF13 expression increased after Clo treatment with and without DEX stimulation (*Figure* *8* *A*). In addition, Clo inhibited the DEX‐induced upregulation of MAFBX and MURF‐1 and downregulation of MYHC in C2C12 cells (*Figure* *8* *A*). Next, a DEX treatment model was used to investigate the effects of Clo on myotube atrophy. Similar to the C2C12 cell results, Clo intervention increased the KLF13 protein level in the skeletal muscle of mice treated with DEX (*Figure* *8* *B*). Mice treated with Clo also had decreased MURF‐1 and MAFBX protein levels after DEX treatment (*Figure* *8* *B*). Additionally, Clo‐treated mice were less prone to the loss of Gas muscle mass upon a DEX challenge based on the body and muscle weights in these mice compared with the vehicle‐treated mice (*Figure* *8* *C,D*). Clo intervention also reversed the DEX‐induced decreased grip strength and exhaustive running distance (*Figure* *8* *E*). Finally, histochemical analysis of the Gas muscle indicated that Clo treatment elicited protective effects against DEX‐induced muscle atrophy based on the markedly larger cross‐sectional diameter of the muscle fibres compared with those from the vehicle treatment group (*Figure* *8* *F*). Collectively, these results confirm that Clo intervention rescues DEX‐induced muscle atrophy.

## Discussion

This study demonstrated that the transcription factor KLF13 plays a multifaceted protective role against GC and CDDP‐induced skeletal muscle atrophy. KLF13 expression was downregulated in several muscle atrophy mouse models, including diabetic, DEX‐treated and cisplatin‐induced models (*Figure* *1* *A,B*), suggesting that KLF13 may be a convergence point for catabolic signalling across different muscle atrophy stimuli and might contribute to muscle atrophy diseases. We also established a causal role of KLF13 in muscle atrophy inhibition. Specifically, *KLF13* overexpression prevented DEX‐mediated atrophy and myotube breakdown in cultured C2C12 cells (*Figure* *5*). Conversely, *KLF13* knockout mice exhibited exacerbated muscle wasting and weakness in response to DEX treatment (*Figure* *2*). Skeletal muscle‐specific *KLF13* overexpression also markedly alleviated diabetes‐associated muscle loss and improved the strength of diabetic mice (*Figure* *3*).

In addition, we mechanistically delineated the pathways through which KLF13 restricted atrophy signalling (*Figures* *4*, *6* *and* *7*). As a transcriptional repressor, we found that KLF13 directly binds to and inhibits the Dll4 expression (a Notch ligand), which is a newly recognized muscle atrophy mediator (*Figure* *4*).^4^ A previous study found that Dll4 derived from endothelial cells bound to Notch2 receptors on muscle fibres in a paracrine manner, activating Notch2 signalling and inducing muscle atrophy without direct contact.^4^ Actually, other studies showed that skeletal muscle fibres can also express and secrete DLL4.^31^, ^32^ In agreement with this, in current study our results demonstrated that the muscle‐derived Notch ligand, Dll4, plays a critical role in muscle atrophy by activating Notch signalling in an autocrine manner. Dll4 is specifically upregulated in the muscle cells of mice of various muscle atrophy models. KLF13 inhibited Dll4 expression and muscle atrophy, likely by directly binding to the Dll4 promoter. These findings provide new insights into the cell‐autonomous Notch‐Dll4 signalling pathway in skeletal muscle atrophy.

Other Notch ligands may also play distinct roles in muscle physiology. Dll1 helps maintain muscle progenitor cells during muscle development, and its loss causes severe muscle hypotrophy.^33^, ^34^ Jag1 maintains muscle stem cell quiescence by crosstalk with Wnt signalling,^35^, ^36^, ^37^ and Jag2 regulates the proliferation and differentiation of muscle stem cells.^38^ Differential Dll3 expression patterns also exist in embryonic muscle development.^39^ Importantly, we found that *KLF13* knockdown increased DLL4 expression and exacerbated muscle atrophy, whereas *KLF13* overexpression suppressed DLL4 and attenuated muscle atrophy. In addition, we found that there is no classical binding element of KLF13 in the promoter of other Notch ligands such as Dll1, Jag1, or Jag2. This suggests that KLF13 specifically targets DLL4 to modulate muscle atrophy, but does not target other Notch ligands. Therefore, we suggest that KLF13 specifically regulates Dll4 to mediate muscle atrophy.

TGF‐β signalling is one mechanism that drives muscle atrophy.^40^, ^41^ TGF‐β levels are elevated in several models of muscle wasting, and TGF‐β signalling inhibition attenuates atrophy.^40^, ^41^ The catabolic effects of TGF‐β are mediated, in part, by stimulating ubiquitin proteasome function, increasing oxidative stress, and suppressing satellite cell differentiation.^40^, ^42^, ^43^ TGF‐β also induces the transcription of MURF‐1, resulting in enhanced protein degradation in muscle atrophy and kidney fibrosis.^44^ Therefore, targeting TGF‐β is an area of active investigation for treating muscle wasting conditions. Our previous work found that KLF13 and its pharmacological agonist, Clo, inhibit TGF‐β activity and prevent fibrosis in kidney diseases.^15^ In this study, we found that KLF13 suppresses TGF‐β signalling by directly inhibiting the transcription of TGF‐β target genes such as alpha‐smooth muscle actin (i.e., *α‐SMA*), collagen type 1 alpha 1 and 2 (i.e., *COL1A1* and *COL1A2*), and fibronectin 1 (i.e., *FN1*).^15^ Thus, KLF13 might mitigate muscle atrophy by inhibiting TGF‐β signalling, which will be explored in the future.

One limitation of our study is that we did not generate skeletal muscle cell‐specific KLF13 knockout mice to observe its impact on DEX‐induced muscle atrophy, and these observations will be conducted in future studies. A second limitation is that we did not investigate whether Clo could improve dexamethasone‐induced muscle atrophy under conditions of KLF13 knockout.

In conclusion, this study highlights the pivotal role of KLF13 as a key regulator of skeletal muscle atrophy by coordinating various sarcopenia triggers. KLF13 inhibits DLL4 transcription and suppresses Notch2 signalling in skeletal muscles, thus, emerging as a central factor in mitigating muscle loss. These discoveries open the door to potential therapeutic approaches to boost KLF13 expression or activity, which could be a promising treatment option for sarcopenia.

## Conflict of interest statement

The authors have declared that no conflict of interest exists.

## Supporting information


**Figure S1.** **KLF13 was reduced in mouse models of muscle atrophy.** (A) Representative image of haematoxylin and eosin (H&E) staining of cross‐sectioned myofibres isolated from mouse tibialis anterior (TA) muscles. The images were captured using a microscope with a × 20 objective. (Below) Cross‐sectional area (CSA) of Gas muscles from mice, *n* = 3 (scale bar = 100 μM). (B) Immunoblot analysis of KLF13 proteins (left) and qPCR analysis of *KLF13* mRNA (right) in TA muscles from diabetic mice (STZ + HFD), wild type (WT) mice treated with DEX or CDDP. (Middle) Quantification represents the levels of the indicated protein normalized to Tubulin, *n* = 6.
**Figure S2. Knockout of KLF13 exacerbated CDDP‐induced muscle atrophy in mice**. We established a CDDP‐induced muscle atrophy model in both WT and Klf13KO mice. **(A)** Immunoblots analysis of KLF13, MAFBX and MURF‐1 using Gas muscle. (Right) Quantification represents the levels of the indicated protein normalized to Tubulin, *n* = 6. **(B)** Total body weight of mice, *n* = 6. **(C)** The ratio of Gas muscle weight and TA muscle weight to body weight, *n* = 6. **(D)** Grip strength test, *n* = 6. **(E)** Representative images of myo cross‐sections were obtained through H&E staining. (Right) Cross‐sectional area (CSA) of Gas muscles from mice, *n* = 6 (scale bar = 100 μM). Data are expressed as means ± SD. In (A, C, D): *P < 0.05, **P < 0.01, ***P < 0.001, by one‐way ANOVA with Bonferroni correction. In (B, E): *P < 0.05, **P < 0.01, ***P < 0.001, by two‐way ANOVA with Bonferroni correction.
**Figure S3. KLF13 overexpression alleviated glucocorticoids‐induced muscle atrophy in mice.** We established a DEX‐induced muscle atrophy model in both WT and K13OE mice. (A) Immunoblots analysis of KLF13, MAFBX and MURF‐1 in Gas muscle. (Right) Quantification represents the levels of the indicated protein normalized to Tubulin, *n* = 6. (B) Total body weight of mice, *n* = 6. (C) The ratio of Gas muscle weight to total body weight, *n* = 6. (D) Exhaustive running distance, *n* = 6. (E) Representative image of H&E staining of myofibre cross‐section from mice. (Right) Cross‐sectional area (CSA) of Gas muscles from mice, *n* = 6 (scale bar = 100 μM). Data are expressed as means ± SD. In (A, C, D, F): *P < 0.05, **P < 0.01, ***P < 0.001, by unpaired Student's t test. In (B): *P < 0.05, by two‐way ANOVA with Bonferroni correction.fiber.
**Figure S4. KLF13 inhibited the expression of DLL4 in the skeletal muscle of mice.** (A,B) Immunoblot analysis of DLL4 and tubulin in Gas from WT or K13KO mice treated with DEX (A) or CDDP (B) as indicated. (Right) Quantification represents the levels of the indicated protein normalized to Tubulin and qPCR analysis was used to detect mRNA levels of Dll4, *n* = 6. (C) Immunoblot analysis of DLL4 and tubulin in Gas from WT or KLF13 overexpression (K13OE) mice using a diabetes‐induced model with STZ and HFD. (Right) Quantification represents the levels of the indicated protein normalized to Tubulin and qPCR analysis was used to detect mRNA levels of Dll4, *n* = 6. Data are expressed as means ± SD. In (A, B): *P < 0.05, **P < 0.01, ***P < 0.001, by one‐way ANOVA with Bonferroni correction. In (C): *P < 0.05, **P < 0.01, CDDP+K13KO vs CDDP, by unpaired Student's t test.
Table S1 Antibodies Information.
Table S2 The sequences of primers for qPCR analysis.
Table S3 PCR primers used for construction of *DLL4 and KLF13* promoters.
Table S4 Primers used in ChIP assays *in vivo*.

**Data S1.** Supporting Information.
