# Supplementary material for: KLF13 restrains Dll4‐muscular Notch2 axis to improve the muscle atrophy
Source: J Cachexia Sarcopenia Muscle. 2024 Jul 8;15(5):1869–82. doi: 10.1002/jcsm.13538 (PMC11446702; doi:10.1002/jcsm.13538)
Supplement: Supplementary file 1 — Figure S1. KLF13 was reduced in mouse models of muscle atrophy. (A) Representative image of haematoxylin and eosin (H&E) staining of cross‐sectioned myofibres isolated from mouse tibialis anterior (TA) muscles. The images were captured using a microscope with a × 20 objective. (Below) Cross‐sectional area (CSA) of Gas muscles from mice, n = 3 (scale bar = 100 μM). (B) Immunoblot analysis of KLF13 proteins (left) and qPCR analysis of KLF13 mRNA (right) in TA muscles from diabetic mice (STZ + HFD), wild type (WT) mice treated with DEX or CDDP. (Middle) Quantification represents the levels of the indicated protein normalized to Tubulin, n = 6. Figure S2. Knockout of KLF13 exacerbated CDDP‐induced muscle atrophy in mice. We established a CDDP‐induced muscle atrophy model in both WT and Klf13KO mice. (A) Immunoblots analysis of KLF13, MAFBX and MURF‐1 using Gas muscle. (Right) Quantification represents the levels of the indicated protein normalized to Tubulin, n = 6. (B) Total body weight of mice, n = 6. (C) The ratio of Gas muscle weight and TA muscle weight to body weight, n = 6. (D) Grip strength test, n = 6. (E) Representative images of myo cross‐sections were obtained through H&E staining. (Right) Cross‐sectional area (CSA) of Gas muscles from mice, n = 6 (scale bar = 100 μM). Data are expressed as means ± SD. In (A, C, D): *P < 0.05, **P < 0.01, ***P < 0.001, by one‐way ANOVA with Bonferroni correction. In (B, E): *P < 0.05, **P < 0.01, ***P < 0.001, by two‐way ANOVA with Bonferroni correction. Figure S3. KLF13 overexpression alleviated glucocorticoids‐induced muscle atrophy in mice. We established a DEX‐induced muscle atrophy model in both WT and K13OE mice. (A) Immunoblots analysis of KLF13, MAFBX and MURF‐1 in Gas muscle. (Right) Quantification represents the levels of the indicated protein normalized to Tubulin, n = 6. (B) Total body weight of mice, n = 6. (C) The ratio of Gas muscle weight to total body weight, n = 6. (D) Exhaustive running distance, n = 6. (E [file JCSM-15-1869-s002.docx]

**Electronic supplementary material**

**ESM Figure and Figure legend**


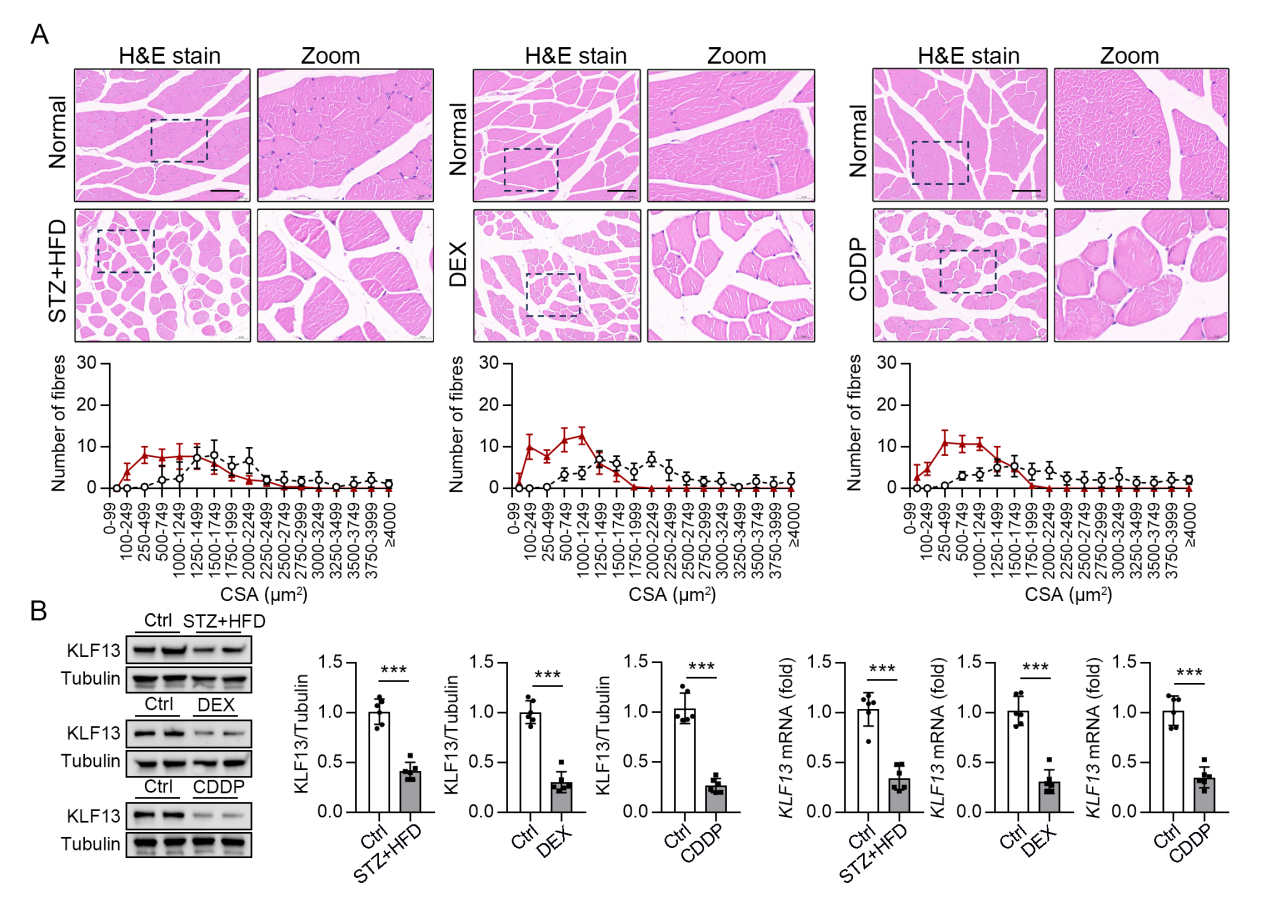


**ESM Figure 1. KLF13 was reduced in mouse models of muscle atrophy.** (A) Representative image of haematoxylin and eosin (H&E) staining of cross-sectioned myofibres isolated from mouse tibialis anterior (TA) muscles. The images were captured using a microscope with a ×20 objective. (Below) Cross-sectional area (CSA) of Gas muscles from mice, n = 3 (scale bar = 100 μM). (B) Immunoblot analysis of KLF13 proteins (left) and qPCR analysis of *KLF13* mRNA (right) in TA muscles from diabetic mice (STZ+HFD), wild type (WT) mice treated with DEX or CDDP. (Middle) Quantification represents the levels of the indicated protein normalized to Tubulin, n = 6.


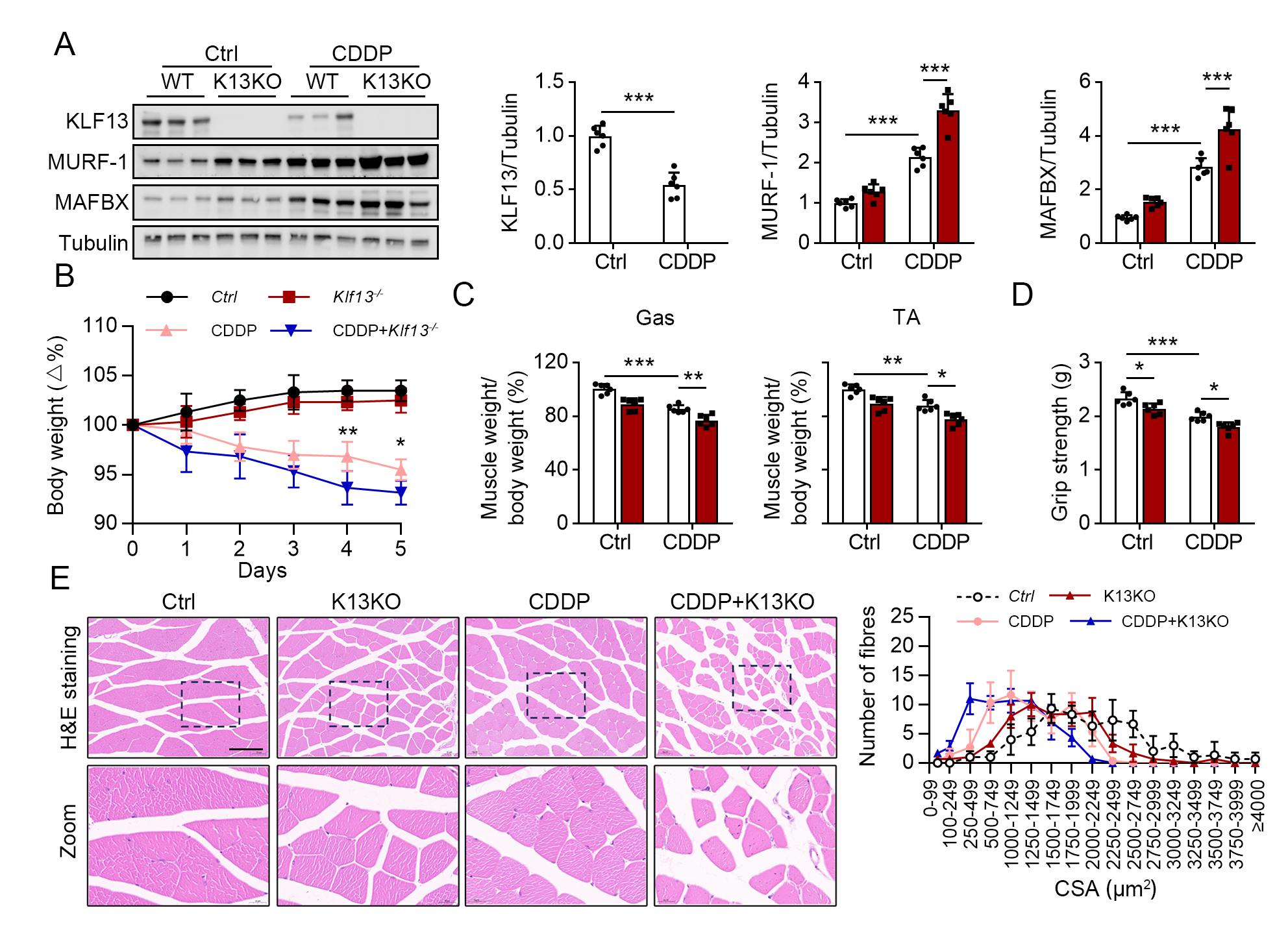


**ESM Figure 2. Knockout of KLF13 exacerbated CDDP-induced muscle atrophy in mice.** We established a CDDP-induced muscle atrophy model in both WT and Klf13KO mice. **(A)** Immunoblots analysis of KLF13, MAFBX and MURF-1 using Gas muscle. (Right) Quantification represents the levels of the indicated protein normalized to Tubulin, n = 6. **(B)** Total body weight of mice, n = 6. **(C)** The ratio of Gas muscle weight and TA muscle weight to body weight, n = 6. **(D)** Grip strength test, n = 6. **(E)** Representative images of myofiber cross-sections were obtained through H&E staining. (Right) Cross-sectional area (CSA) of Gas muscles from mice, n = 6 (scale bar = 100 μM). Data are expressed as means ± SD. In (A, C, D): **P* < 0.05, ***P* < 0.01, ****P* < 0.001, by one-way ANOVA with Bonferroni correction. In (B, E): **P* < 0.05, ***P* < 0.01, ****P* < 0.001, by two-way ANOVA with Bonferroni correction.


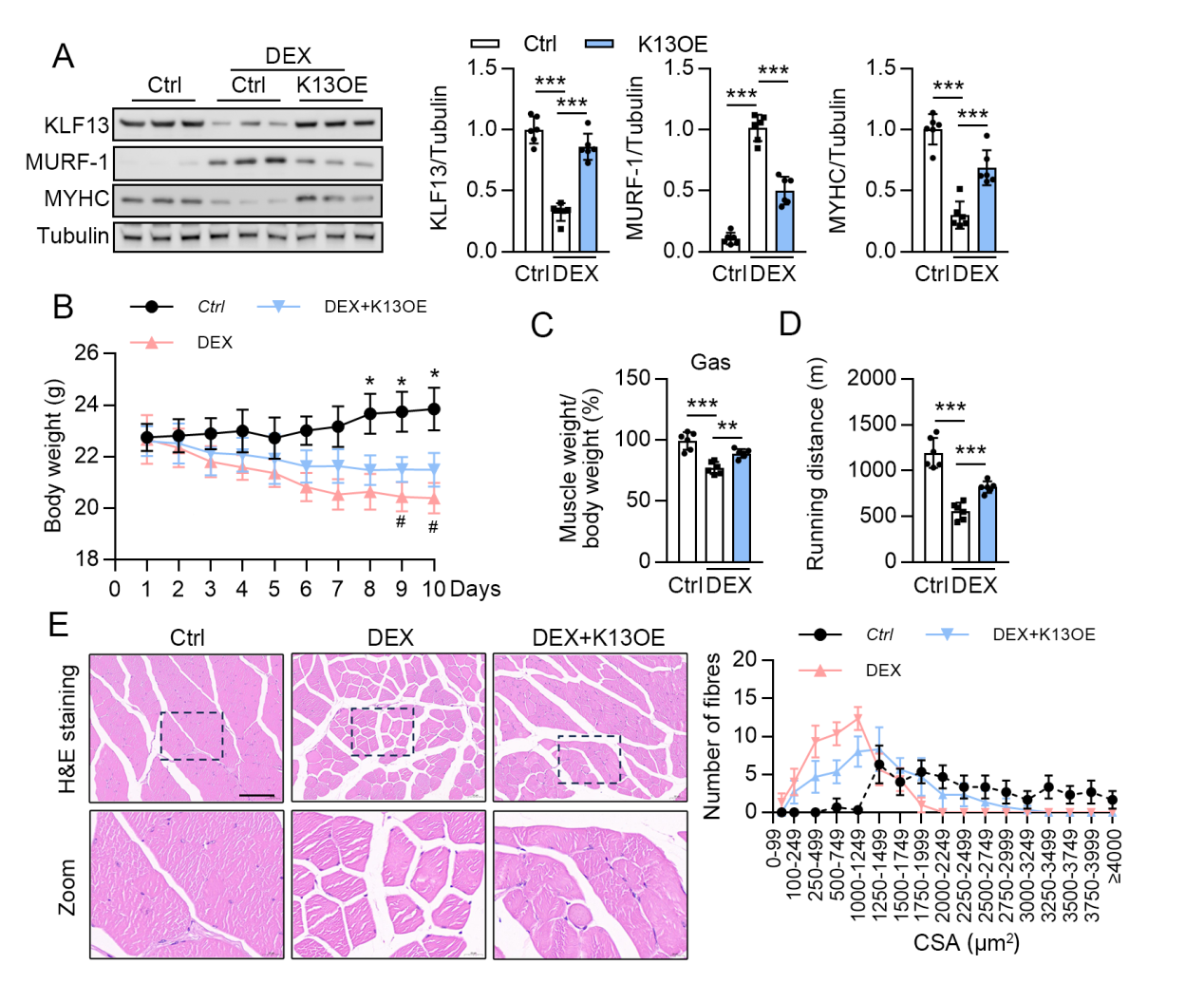


**ESM Figure 3. KLF13 overexpression alleviated glucocorticoids-induced muscle atrophy in mice.** We established a DEX-induced muscle atrophy model in both WT and K13OE mice. (A) Immunoblots analysis of KLF13, MAFBX and MURF-1 in Gas muscle. (Right) Quantification represents the levels of the indicated protein normalized to Tubulin, n = 6. (B) Total body weight of mice, n = 6. (C) The ratio of Gas muscle weight to total body weight, n = 6. (D) Exhaustive running distance, n =6. (E) Representative image of H&E staining of myofibre cross-section from mice. (Right) Cross-sectional area (CSA) of Gas muscles from mice, n = 6 (scale bar = 100 μM). Data are expressed as means ± SD. In (A, C, D, F): *P < 0.05, **P < 0.01, ***P < 0.001, by unpaired Student’s t test. In (B): *P < 0.05, by two-way ANOVA with Bonferroni correction.


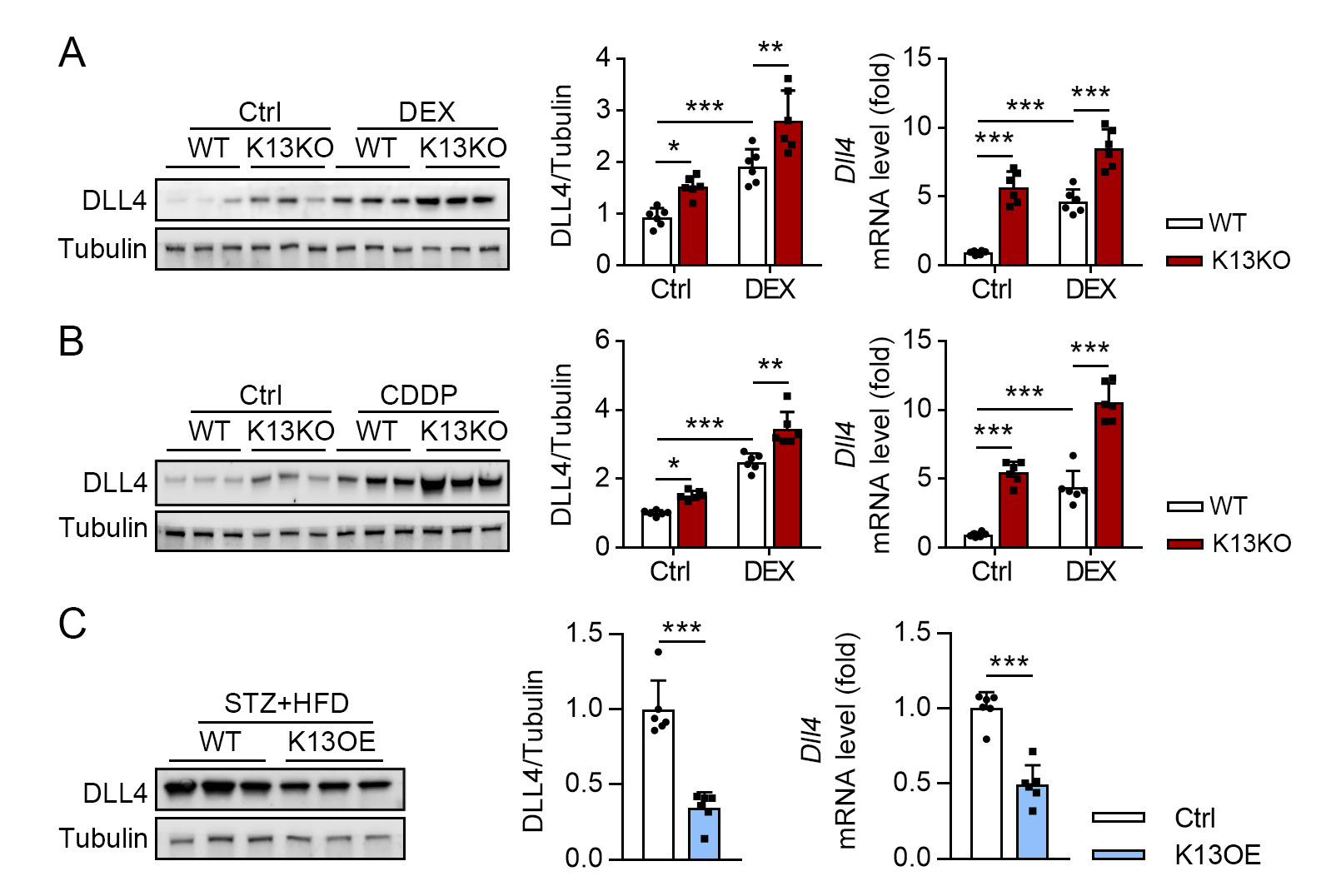


**ESM Figure 4. KLF13 inhibited the expression of DLL4 in the skeletal muscle of mice.** (A,B) Immunoblot analysis of DLL4 and tubulin in Gas from WT or K13KO mice treated with DEX (A) or CDDP (B) as indicated. (Right) Quantification represents the levels of the indicated protein normalized to Tubulin and qPCR analysis was used to detect mRNA levels of Dll4, n = 6. (C) Immunoblot analysis of DLL4 and tubulin in Gas from WT or KLF13 overexpression (K13OE) mice using a diabetes-induced model with STZ and HFD. (Right) Quantification represents the levels of the indicated protein normalized to Tubulin and qPCR analysis was used to detect mRNA levels of Dll4, n =6. Data are expressed as means ± SD. In (A, B): **P* < 0.05, ***P* < 0.01, ****P* < 0.001, by one-way ANOVA with Bonferroni correction. In (C): **P* < 0.05, ***P* < 0.01, CDDP+K13KO vs CDDP, by unpaired Student’s t test.

**ESM Methods**

**Prediction of Transcription Factors**

JASPAR (http://jaspar.genereg.net/) is an open-access database containing manually curated, non-redundant transcription factor (TF) binding profiles for TFs across six taxonomic groups. The promoter region of the target gene was obtained from UCSC (<http://genome.ucsc.edu/>).

**Protein stability assay**

The stability of KLF13 proteins was measured using a cycloheximide pulse chase assay as previously described[15]. In brief, C2C12 cells were treated with 50 μg/mL cycloheximide to block protein translation. KLF13 protein levels were quantified with immunoblotting at 0, 15, 30, 60, 90, 120 min after cycloheximide treatment and expressed as percentages relative to those at time 0.

**Dual-luciferase reporter assay**

The full-length human *KLF13* and *DLL4* genes from HEK293T cells (human embryonic kidney cell line) cDNA were amplified by PCR. Fragments of the target gene promoter were cloned by PCR and inserted into the pGL3 luciferase vector using the primers listed in ESM Table 3. All constructs were confirmed by DNA sequencing analysis. For dual luciferase reporter gene assays, HEK293T cells were transfected with the target gene promoter plasmids and KLF13 as well as Renilla luciferase. Firefly and Renilla luciferase activities were then measured by a dual luciferase reporter gene system (Promega, Madison, WI, USA).

**Chromatin immunoprecipitation (ChIP) and sequential ChIP assay**

ChIP assays were performed with a commercial kit from Sigma-Aldrich (St. Louis, Missouri, USA) following the manufacturer’s instructions with the primers listed in ESM Table 4. Briefly, liver tissues were lysed in lysis buffer and sonicated (15 s on and 90 s off, repeated eight times). After precipitation with Agarose A for 30 min, the fragmented DNA was pulled down with KLF13 and MYOD1 antibodies or IgG and then subjected to amplification by qPCR.

**Protein extraction and western blot analysis**

Proteins were extracted from C2C12 cells or mouse muscle tissues (Gas) using radioimmunoprecipitation assay buffer (Solarbio Science & Technology) supplemented with protease and phosphatase inhibitors (Thermo Fisher Scientific, Massachusetts, USA). The protein concentration was determined using the Rapid Gold BCA Protein Assay Kit (Thermo Fisher) following the manufacturer’s protocol. For western blot analysis, 50 µg of lysate was loaded onto sodium dodecyl sulfate-polyacrylamide gel electrophoresis gels, and transferred onto polyvinylidene difluoride membranes (Millipore). Proteins were analyzed with their corresponding specific antibodies. Antibody information is listed in the ESM Table 1. Densitometry analysis was performed using Quantity One® Software and quantified relative to the loading control, Tubulin

**Histological analysis**

Muscle tissue was embedded in paraffin and sliced into 5 μm thick serial sections using a paraffin slicer. For muscle histology, the paraffin sections were stained using Mayer’s hematoxylin and eosin staining kit (Solarbio & Technology, Beijing, China).

**Determination of FBG and fasting FBI**

Serum samples from mice that had fasted overnight were utilized to measure blood glucose and insulin levels. Blood glucose levels were assessed using a OneTouch® glucometer and test strips (LifeScan, Milpitas, CA, USA), while insulin levels were measured using an insulin ELISA kit (ab277390; Abcam, Cambridge, MA, USA).

**GTT and ITT**

Glucose tolerance tests (GTTs) and insulin tolerance tests (ITTs) were conducted on mice at 22 and 23 weeks of age, respectively, following previously established protocols[17]. Briefly, the mice were fasted for 6 hours and then administered glucose (2 g glucose/kg body weight; Cat. no. Y0001745, Sigma-Aldrich, St.Louis, MO, USA) via intraperitoneal injection for the GTT assay, or insulin (1 U insulin/kg body weight; Actrapid, Novo Nordisk, Denmark) for the ITT assay. Blood glucose levels were measured at 0, 15, 30, 60, 90, and 120 minutes after injection. Fasting blood glucose levels were determined during the experiment using blood samples collected from the mouse tail vein and analyzed with the OneTouch glucometer and test strips (LifeScan, Milpitas, CA, USA).

**Immunofluorescent staining of C2C12 cells**

After treatment, myofibre immunostaining of C2C12 cells was fixed for 15 min using fresh, methanol-free 4% formaldehyde, and then, rinsed thrice with PBS for 5 min each. After blocking with goat serum for 30 min, the cells were incubated with primary antibody against Myh (Abcam, Cat. No. ab172967, 1:100) at 4 °C overnight. Alexa Fluor®488 goat antibodies against murine IgG (Invitrogen, Shanghai, China; Cat. No. A-11078, 1:400) were included as secondary antibodies. As negative controls, the primary antibodies were exchanged for nonimmune serum from the same species. The samples were counterstained with DAPI for 15 min. The sections were sealed with a cover glass, and the specimens were examined using the appropriate excitation wavelength. Images were captured and processed with a Laica microscope (Wetzlar, Germany).

**Cell culture**

C2C12 myoblasts (Cat. no.: SCSP-505) were obtained from the National Collection of Authenticated Cell Cultures (Shanghai, China). Regular mycoplasma testing was conducted using a qPCR test that was performed under ISO17025 accreditation to ensure the absence of mycoplasma contamination. The C2C12 myoblasts were cultured according to previously established protocols[16]. They were maintained in growth medium consisting of Dulbecco’s Modified Eagle Medium, high glucose (Gibco, Grand Island, NY, USA), supplemented with 10% fetal bovine serum (Gibco), 10 U/ml penicillin, and 10 μg/ml streptomycin (Welgene, Taipei, China), at 37°C with 5% CO2. Differentiation medium consisted of Dulbecco’s Modified Eagle Medium, high glucose, supplemented with 2% horse serum, 10 U/ml penicillin, and 10 μg/ml streptomycin. Prior to treatment, C2C12 myoblasts were cultured in differentiation medium for 3 days.

**Grip Strength Test**

A digital grip strength meter was utilized for assessing the forelimb grip strength of mice. The mice were gently lifted by their tails and their forelimbs were positioned on the grip lever. Then, they were smoothly pulled away from the transducer in a horizontal plane, and the maximum force exerted on the transducer was recorded. This procedure was repeated for three sets, with each set consisting of ten repetitions. A 15-minute rest period was provided between each set. The highest effort exerted by the mouse during each attempt was recorded and designated as the maximum grip strength.

**Exhaustive running distance test**

The mice were positioned on the treadmill (Jiangsu SANS Biological Technology Co. Ltd, China), and the shock grid was activated throughout the entire experiment with 0° incline. The speed of the treadmill was gradually increased until the mice reached exhaustion, as depicted in Figures 3 and 5. Exhaustion was determined as the point at which the mice maintained continuous contact with the shock grid for a duration of 5 seconds.

**Coimmunoprecipitation (Co-IP)**

After treatment, the cells were lysed in an ice-cold co-immunoprecipitation (co-IP) buffer containing 20 mM Tris-HCl (pH 8.0), 100 mM NaCl, 1 mM EDTA, and 0.5% NP-40, supplemented with a protease inhibitor cocktail (Roche, 04693132001), for 30 min. The cell homogenates were then centrifuged at 13,000g for 15 min, and the resulting supernatant was incubated overnight at 4 °C on a shaker with primary antibodies [anti-KLF13 (Novus, Cat. No. NBP2-98809), anti- FBXW7 (Novus, Cat. No. NBP2-50403) and anti-IgG]. To ensure complete saturation of the primary antibodies, sufficient cell lysates were cultured and collected for immunoprecipitation (IP). The mixture of antibodies and proteins was subsequently incubated with protein A/G-agarose beads (Thermo Fisher Scientific, Cat. No. 78610) at 4 °C for 3 h. The beads were washed 5-6 times with cold IP buffer and resuspended in loading buffer. The cell lysates and immunoprecipitates were denatured in loading buffer at 95 °C for 5 min, and western blotting analysis was performed.

**ESM Table**

**Table 1** **Antibodies Information**

| Antibodies | Source | Catalog No. |
| --- | --- | --- |
| Rabbit Polyclonal antibody anti- KLF13 | Proteintech | Cat# 18352-1-AP |
| Mouse monoclonal anti-MYHC | R&D | Cat# MAB4470 |
| Mouse monoclonal anti-MAFBX (F-9) | Santa Cruz Biotechnology | Cat# SC166806 |
| Rabbit monoclonal anti-β-Tubulin | Proteintech | Cat# 10094-1-AP |
| Mouse monoclonal anti-MURF-1 | Santa Cruz Biotechnology | Cat# sc-398608 |
| Mouse Polyclonal anti-DLL4 | R&D | Cat# AF1389 |
| Mouse monoclonal anti-Ubiquitin | Novus | Cat# NB300-130 |
| Rabbit monoclonal anti-MYOD1 | abcam | Cat# ab203383 |
| Rabbit polyclonal anti-FBXW7 | Novus | Cat# NBP1-59631 |

**Table 2 The sequences of primers for qPCR analysis**

| Gene | Forward | Backward |
| --- | --- | --- |
| *mus-Dll4*  (ID: 54485) | TTCCAGGCAACCTTCTCCGA | ACTGCCGCTATTCTTGTCCC |
| *mus-Jag1*  (ID: 16449) | CCTCGGGTCAGTTTGAGCTG | CCTTGAGGCACACTTTGAAGTA |
| *mus-Dtx3*  (ID: 80904) | ACCCAATGTCATCACTTGGAAC | CCTCTTGCACCCTAGTCAGGT |
| *mus-Psen2β*  (ID: 19165) | GAAGACTCCTACGACAGTTTTGG | CACCAGGACGCTGTAGAAGAT |
| *mus-Dtx4*  (ID: 207521) | TGTGCCTGTGAAAAACTTGAATG | TGGGATGGACTTTATCTCACTCT |
| *mus-Dll1*  (ID: 13388) | CAGGACCTTCTTTCGCGTATG | AAGGGGAATCGGATGGGGTT |
| *mus-Ccng2*  (ID: 12452) | AGGGGTTCAGCTTTTCGGATT | AGTGTTATCATTCTCCGGGGTAG |
| *mus-Cdkn1b*  (ID: 12576) | TCAAACGTGAGAGTGTCTAACG | CCGGGCCGAAGAGATTTCTG |
| *mus-Rbl2*  (ID: 19651) | AACTTCCCCATGATTAGCGATG7 | GGTTAGAACACTGAAGGGCATTT |
| *mus-Bnip3*  (ID: 12176) | TCCTGGGTAGAACTGCACTTC | GCTGGGCATCCAACAGTATTT |
| *mus-Fbxo32*  (ID: 67731) | CAGCTTCGTGAGCGACCTC | GGCAGTCGAGAAGTCCAGTC |
| *mus-Fbxo30*  (ID: 71865) | TATGAACTGTGTCAGTAGACGGT | CGATGTTCGTCAGCTTTACAAGA |
| *mus-Fbxo31*  (ID: 76454) | CATGCGGTTCAAGCCACTG | GTCTGGTTACACTTGGTGGAG |
| *mus-actin*  (ID: 11461) | GGCTGTATTCCCCTCCATCG | CCAGTTGGTAACAATGCCATGT |

**Table 3 PCR primers used for construction of *DLL4 and KLF13* promoters**

| *DLL4* | Forward Primer (5’→3’) | Reverse Primer (5’→3’) |
| --- | --- | --- |
| -3123 Luc | (CGGGGTACC)TATGGCTGGAGGACTATGCG | (TCCCCCGGG)CGCTCGTTGATGAACTCCTG |
| -2236 Luc | (CGGGGTACC)GGAGTTGAGAGAGGACAGGG | (TCCCCCGGG)CGCTCGTTGATGAACTCCTG |
| -1344 Luc | (CGGGGTACC)CCTTGATTCTCCAGTGGCCT | (TCCCCCGGG)CGCTCGTTGATGAACTCCTG |
| -380 Luc | (CGGGGTACC)GCGAGGAGAGGAGCCAATAT | (TCCCCCGGG)CGCTCGTTGATGAACTCCTG |
| *KLF13* | Forward Primer (5’→3’) | Reverse Primer (5’→3’) |
| -3302 Luc | (CGGGGTACC)AAAGGGGTTTGTGGGTCTCA | (TCCCCCGGG)GCTGGTTGAGGTCCGCTA |
| -2257 Luc | (CGGGGTACC)GCCACAGAGCACAGTCAAAA | (TCCCCCGGG)GCTGGTTGAGGTCCGCTA |
| -1380 Luc | (CGGGGTACC)GCTGGTGGCGTTTGAAGC | (TCCCCCGGG)GCTGGTTGAGGTCCGCTA |
| -351 Luc | (CGGGGTACC)GTCCCGCCTGCCACAATG | (TCCCCCGGG)GCTGGTTGAGGTCCGCTA |

**Table 4 Primers used in ChIP assays *in vivo***

| Target gene *in vivo* | Target sequence |
| --- | --- |
| *KLF13* MBE | Forward: 5’ - TACCATACAGCTGAGGACGC -3’ |
|  | Reverse: 5’ - CTAAGGGTCTAAGGCTCGGG -3’ |
| *DLL4* KLFE | Forward: 5’ - GTGGCCCAAGGAAATTCTGG -3’ |
|  | Reverse: 5’ - GCCAGGACTAGAGGTTCGAA -3’ |
